# Men. Male-biased sex ratios and masculinity norms: evidence from Australia’s colonial past

**DOI:** 10.1007/s10887-023-09223-x

**Published:** 2023-04-01

**Authors:** Victoria Baranov, Ralph De Haas, Pauline Grosjean

**Affiliations:** 1 Department of Economics, University of Melbourne, Melbourne, Australia; 2 European Bank for Reconstruction and Development, London, UK; 3 UNSW, Sydney, Australia; 4 CEPR, London, UK; 5 KU Leuven, Leuven, Belgium

**Keywords:** Masculinity, Identity, Sex ratio, Natural experiment, Cultural persistence, I31, J12, J16, N37, O10, Z13

## Abstract

We document the historical roots and contemporary consequences of masculinity norms—beliefs about the proper conduct of men. We exploit a natural experiment in which convict transportation in the $18^{\textrm{th}}$ and $19^{\textrm{th}}$ centuries created a variegated spatial pattern of sex ratios across Australia. We show that in areas with heavily male-biased convict populations, relatively more men volunteered for World War I about a century later. Even at present these areas remain characterized by more violence, higher rates of male suicide and other forms of preventable male mortality, and more male-stereotypical occupational segregation. Moreover, in these historically male-biased areas, more Australians recently voted against same-sex marriage and boys—but not girls—are more likely to be bullied in school. We interpret these results as manifestations of masculinity norms that emerged due to intense local male-male competition. Once established, masculinity norms persisted over time through family socialization as well as peer socialization in schools.

**Supplementary Information:**

The online version contains supplementary material available at 10.1007/s10887-023-09223-x.

## Introduction

What makes a ‘real’ man? A particular normative form of masculinity, often described as hegemonic, posits that men ought to be self-reliant, assertive, competitive, dominant, violent when needed, and in control of their emotions (Mahalik et al., 2003; Connell & Messerschmidt, 2005). Three current debates illustrate how such masculinity norms can have profound economic and social impacts. A first debate concerns the fact that in many countries men die younger than women, and are consistently less healthy (Case & Paxson, 2005; IHME, 2010; Baker et al., 2014). Masculinity norms—especially a penchant for violence and risk taking—are an important cultural driver of this gender health gap (WHO, 2013; Schanzenbach et al., 2016).

A second debate links masculinity norms to occupational gender segregation. Technological progress and globalization have disproportionately affected male employment (Autor et al., 2019). Many newly unemployed men nevertheless refuse to fill jobs that do not match their self-perceived gender identity (Akerlof & Kranton, 2000, 2010) and choose instead to remain unemployed or leave the labor force (Katz, 2014). Restrictive masculinity norms then impose constraints on occupational choice that may be economically inefficient if they increase search costs, misallocate talent, and lead to sectoral staff shortages. Economic growth may suffer as a result.

Third, masculinity norms have become integral to debates about the socio-economic inclusion of women and sexual minorities in Western society. These cultural changes can threaten the identity of men who adhere to conservative masculinity norms, provoking a backlash against women and minorities (Kimmel, 2013; Horvilleur, 2019; Inglehart & Norris, 2019).

While there are striking similarities across countries regarding the ideals that men are expected to adhere to (Gilmore, 1990), the extent to which men have to conform to such norms differs across societies (Traister, 2000). This raises the question: Where do masculinity norms come from? The origins of gender norms that guide and constrain the behavior of *women* have been the focus of an important recent literature (Fernández et al., 2004; Alesina et al., 2013; Carranza, 2014; Giuliano, 2018; Grosjean & Khattar, 2019; Galor et al., 2020). Our focus is, instead, on the origins and manifestations of norms that guide and constrain the behavior of *men*.

Our contribution is to show how masculinity norms can be shaped by historical circumstances that skew sex ratios, thus creating a shortage of women and heightening competition among men. Intense male-male competition not only establishes a dominance order (that is, it determines males’ relative access to resources and mates) but also gives rise to a set of behavorial norms.

To establish a causal link from sex ratios to the manifestation of masculinity norms, we exploit a natural experiment—the convict colonization of Australia. Between 1787 and 1868, Britain transported 132,308 convict men but only 24,960 convict women to Australia. This imposed a variegated spatial pattern in sex ratios, which led to local variation in male-to-male competition in an otherwise homogeneous setting. We test this idea by combining information on historical sex ratios among convicts, using data from Australian colonial censuses compiled by Grosjean and Khattar (2019) [henceforth GK], with proxies for intermediary and present-day masculinity norms. These include voluntary recruitment during WWI, violent behavior and crime, suicide, bullying, help-avoiding behavior, COVID-19 vaccine hesitancy, and stereotypically male occupational choice. Moreover, we capture the political expression of masculine identity by opposition against same-sex marriage, which we measure using voting records from a unique nation-wide referendum on same-sex marriage in 2017.

We focus on these outcomes as they are well-accepted behavioral manifestations of hegemonic masculinity norms in Western societies.^1^ Mahalik et al. (2003) develop an inventory of 11 core masculinity norms: winning; emotional control; risk-taking; violence; dominance; playboy; self-reliance; primacy of work; power over women; disdain for homosexuals; and pursuit of status.^2^ Among these, we focus on those norms that are likely to generate behaviors that are observationally distinct from behaviors that are influenced by male–female bargaining. After all, certain behaviors classified as manifestations of masculinity norms, such as the primacy of work, pursuit of status, or power over women, may also be influenced by male–female bargaining (with male-biased sex ratios granting a more favorable bargaining position to women). This may make it difficult to single out masculinity norms as a separate channel.

For this reason, this paper studies behavioral manifestations of masculinity norms for which the conditions of male–female bargaining should either have no influence or select for *opposite* behaviors. The most prominent example is male violence. Men who are behaviorally aggressive towards other men in competitive contexts may also be prone to aggression in the context of marriage or other long-term relationships. They may also be prone to sexual coercion.^3^ Studies show that women have a distaste for violent men and turn away from men whose traits signal aggressive potential (Li et al., 2014). More generally, women tend to prefer cooperative men (Phillips et al., 2008).

Other behavioral manifestations of masculinity norms that we study in this paper—such as help-avoiding behavior (and associated premature death), male suicide, and voluntary participation in WWI—negatively affect women as wives, especially in an environment where men are economic providers. These behaviors, as well as the bullying of boys in schools and low tolerance of same-sex relationships, also hurt mothers (possibly more than fathers, to the extent that women care more for their children’s welfare^4^). We check that the proxies for masculinity norms that we use in this paper are uncorrelated with gender norms about the social and economic role of women.

Our results paint a consistent picture of how skewed sex ratios instilled masculinity norms that deeply influence the social and economic landscape to this day. By way of preview, we find that a one standard deviation increase in the sex ratio among convicts is associated with a 5.6% increase in the share of men who served in WWI, with no effect on female volunteers. Areas that were more male-biased in the past (though not the present) remain characterized by more assaults (+ 8.8%), sexual assaults (+ 12.8%), male suicide rates (+ 20.2%), prostate cancer (+ 3.3%), and COVID-19 vaccine hesitancy among men (+18.2 percent). A one standard deviation increase in the convict sex ratio is also associated with a 0.7% point shift in the share of men employed in feminine or neutral occupations towards stereotypically male occupations.

Moreover, we find that in areas that were heavily male-biased, fewer Australians support same-sex marriage today, and boys are more likely to fall victim to bullying in school. A one standard deviation increase in the convict sex ratio is associated with a 2.2% point decrease in the probability of voting "Yes" to same-sex marriage and a 3.6–8.5% point increase in the bullying of boys in schools, depending on whether we base our estimates on reports by teachers or parents. We take this last result as evidence of peer socialization and the transmission of masculinity norms, which helps to explain the persistent effects of historical sex ratios. Importantly, we see no variation in the rates of non-violent crime, in political opinions unrelated to the status of sexual minorities, in female suicide, female COVID vaccine hesitancy, or in the bullying of girls.

We interpret these strong local impacts of historical sex ratios on intermediary and present-day outcomes for men as manifestations of hegemonic masculinity norms. We back up this interpretation by bringing additional survey data to bear that reveal a tight relationship between actual measurements of Australian men’s conformity to masculinity norms and outcomes such as suicide attempts; violent behavior; and health care avoidance.

The main empirical challenge in estimating the impact of sex ratios on manifestations of masculinity norms is that variation in sex ratios usually reflects characteristics that arise from spatial selection. Men and women sort across geographic areas based on observable or unobservable characteristics that are possibly related to outcomes of interest. For example, fewer women may choose to live in areas where men are more violent. In turn, such characteristics may persist over time and induce a spurious correlation between historical sex ratios and the type of present-day outcomes that are attributable to masculinity norms. We avoid this confound by focusing on historical sex ratios among convicts. Convicts were not free to move: a centralized assignment scheme determined their location as a function of labor needs to develop the country, which we proxy by initial economic specialization. This circumvents the possibility that our results are driven by self-selection across different areas of Australia.

Throughout, our estimates include state fixed effects to account for the influence of time-invariant state characteristics such as legislation or differences in patterns of settlement across states. In addition, we check that convict sex ratios do not systematically vary as a function of environmental or economic characteristics and are uncorrelated with industrial specialization. Even then, our results are robust to controlling for such initial circumstances, including mineral or land endowments and economic specialization. Our results also hold in a wide range of robustness tests—such as including additional contemporaneous controls like the present-day sex ratio, urbanization, share of various religious groups, and unemployment. Oster (2019) bounds confirm that our estimated coefficients are relatively stable, thus alleviating concerns about omitted variables basis. Moran statistics show that our findings do not merely reflect spatial autocorrelation of the error terms.

A concern is that convicts were different from the rest of the population in ways that are correlated with our outcomes of interest. In particular, convicts may have been more prone to violence, crime, and risk taking and it could be the persistence of this convict ‘stain’ that we observe today.^5^ Historical evidence argues against such a mechanism. As we describe in the historical background section, convicts transported to Australia were not “hardened and professional criminals" (Nicholas, 1988, p. 3) but rather “ordinary working-class men and women" (Nicholas, 1988, p. 7). The majority was transported for a first offense, usually a minor property offense such as petty theft (Oxley, 1996). Nevertheless, we control for the number of convicts, together with total population, throughout.

Our results contribute to several strands of the literature. First and foremost, we provide a new perspective on the causes, nature, and consequences of gender norms (Giuliano, 2018).^6^ Recent work explores the historical origins of norms about women, including differences in technology (Alesina et al., 2013; Xue, 2016), soil structure (Carranza, 2014), political institutions (Lippmann et al., 2016) or sex ratios (Gay 2021, GK, Teso, 2019, Caicedo et al., 2020). Related work assesses the impact of the resulting female identity on household formation and female work choices (Bertrand et al., 2015).

The previous economic literature on the effects of sex ratios has focused on male–female bargaining. In line with models of the marriage market (Becker, 1973, 1974), studies have shown how a relative scarcity of women influences how men and women interact within the household (Heer & Grossbard, 1981; Grossbard-Shechtman, 1984; Chiappori et al., 2002; Grossbard & Amuedo-Dorantes, 2008; Grossbard, 2015). Over time these interactions shape social norms about female work (Gay, 2021; Grosjean & Khattar, 2019). Instead, we focus on a different, and novel, mechanism: how a scarcity of women determines how men interact and compete with *one other* and thus shape behavioral norms for *men*.^7^

We document how such masculinity norms continue to manifest themselves in various ways, such as men shunning stereotypically female occupations, engaging in violence, and opposing same-sex marriage. We put forward intrasexual competition as a theoretical framework to understand the contemporaneous relationships between skewed sex ratios and violent crime (Hesketh & Xing, 2006; Edlund et al., 2013; Cameron et al., 2019), molestation and rape (Ullman & Fidell, 1989), as well as suicide and early mortality (Chowdhry, 2005; Chang et al., 2021), which have been documented in other contexts.^8^ Our results suggest that these relationships may be longer lasting than previously thought if these behaviors become entrenched norms.^9^

We also contribute to an emerging literature on the economic role of norms and identity (Akerlof & Kranton, 2000, 2010; Bénabou & Tirole, 2011; Gennaioli & Tabellini, 2019) as well as stereotypes (Bordalo et al., 2016). Several studies highlight the role of perceived threats to one’s honor or reputation (Nisbett & Cohen, 1996; Cohen et al., 1996; Grosjean, 2014; Cao et al., 2021) or one’s masculinity (Wilson & Daly, 1985) as drivers of violence. We suggest that concerns about status or male identity are heightened in more competitive environments and can have long-lasting effects on violent tendencies towards others but also oneself (suicide).

Relatedly, conforming to hegemonic masculinity norms has been hypothesized to constitute an important cause of stubborn male unemployment despite the availability of (stereotypically female) service jobs (Akerlof & Kranton, 2010; Katz, 2014), thereby potentially lowering economic growth. We provide empirical evidence that masculinity norms can indeed manifest themselves in the labor market through male-stereotypical occupational segregation.

Lastly, we contribute to the literature on the determinants of support for minorities’ civil rights, such as same-sex relationship recognition. Most studies focus on individual correlates of attitudes towards sexual minorities, highlighting the role of gender (Kite, 1984); education and rurality (Stephan & McMullin, 1982; Lottes & Kuriloff, 1994; Herek & Capitanio, 1996); or age and religion (Inglehart, 1990; Edwards, 2007).^10^ A recent paper by Fernández et al. (2021) explores how (media coverage of) political discussions about the ban on gay people in the U.S. military changed attitudes towards same-sex relationships. Our contribution is to uncover historical roots of attitudes towards homosexuality and to suggest masculinity norms as a mechanism through which such attitudes become entrenched. Related to our work, Brodeur and Haddad (2021) find that same-sex relationships are more prevalent in places in the U.S. that experienced a Gold Rush. Their hypothesized mechanism consists of the self-selection of gay men to Gold Rush places, while our setting, based on the quasi-random allocation of British convicts, rules out initial self-selective migration on the basis of sexual preferences. A unique feature of our study is also that the Australian referendum provides unbiased and high-quality data on citizens’ revealed preferences for civil rights for sexual minorities. Given that real legislation was at stake, and turnout was high (at 79.5%), these data arguably better reflect people’s convictions than surveys that have so far been used to elicit attitudes towards sexual minorities.

We proceed as follows. Section 2 describes the conceptual background after which Sect. 3 provides some historical detail about colonial Australia. Section 4 describes the various data. Sections 5 and 6 then discuss our empirical approach and results. Section 7 considers mechanisms and Sect. 8 concludes.

## Conceptual background

This section provides a conceptual discussion of the link between sex ratios and male-male competition (Sect. 2.1) and of the impact of sex ratios on masculinity norms and related outcomes (Sect. 2.2).

### Sex ratios, male-male competition, and male–female bargaining

The sex ratio, the number of males relative to females, is a central concept in evolutionary biology. The idea that behavioral differences between the sexes originate in the conditions of reproductive competition, among which the sex ratio plays a primordial role, is the cornerstone of Darwin’s *The Descent of Man* (1871). When the sex ratio is more male biased, competition between males is more intense. Across a wide range of taxa, strong male-male competition induces risk taking, violence and control, oftentimes exerted through violent means, over the reproductive opportunities of dominated males as well as females (Emlen & Oring, 1977; Buss, 2016). Experimental studies of lizards, birds, and primates find that male-biased sex ratios increase male aggression towards males as well as females (Sapolsky, 1990, 1991). In human populations, skewed sex ratios have likewise been shown to correlate with rape and other violent crime.^11^

Among humans, the behavioral consequences of male-biased sex ratios have so far been mostly analyzed through the lens of male–female bargaining, i.e. *inter*-sexual competition, within the framework of the Beckerian household model. Several contributions have studied how male-biased sex ratios increase female bargaining power and consequently shift resources and family structures. Women are then less likely to participate in the labor force (Grossbard-Shechtman, 1984; Chiappori et al., 2002; Grossbard & Amuedo-Dorantes, 2008; Grossbard, 2015), also work less within the home, and enjoy more leisure as a result (Grosjean & Khattar, 2019). Men, in contrast, work and save more to become attractive partners (Wei & Zhang, 2011) and adopt behavior and mating strategies more favorable to females’ interests (Guttentag & Secord, 1983; Pedersen, 1991). In particular, male-biased sex ratios correlate with more monogamy, more committed relationships and higher marriage rates (Grosjean & Khattar, 2019; Schacht & Kramer, 2016), greater marital stability and satisfaction (Otterbein, 1965; Grosjean & Brooks, 2017), and more paternal involvement (Schmitt, 2005).^12^

To sum up, the literature contrasts the effects of sex ratios on aggression and violence in domains of intra-sexual competition—which have been documented across multiple animal taxa and are the focus of a large evolutionary biology literature—with their effects on inter-sexual cooperation, which has been the primary focus in economics. Alger (2021) develops a theoretical model that brings both elements together by conceptualizing male-male competition and male–female household formation as sequential matching evolutionary game. The strategies in the initial competition stage are the degrees of competitiveness. At stake are women (reproductive resources) and productive (material) resources that enable a man to provide parental care. An implication of this model is that male-biased sex ratios increase male-male competition in the short-run—if there are fewer women than men, not competing is not evolutionary stable—as well as in the long-run, since the degree of competitiveness is transmitted from fathers to sons. Another insight is that the outcome of the male-male competition stage is a *fait accompli* at the stage of female choice, and hence of male–female household bargaining later on. This is because, when faced with the order established by competition, a female’s reproductive success will be higher if she accepts the winner of the competition, who brings in additional resources.

In this paper, we ask what predictions can be made with respect to the influence of sex ratios on human behavior that operate through the mechanism of male-male competition. To do so, we focus on norms and behavioral outcomes for which male-male competition leads to predictions that are unrelated or opposite to the expected effects of male–female bargaining.

### Skewed sex ratios, masculinity norms, and behavioral outcomes: Hypotheses

Because male-biased sex ratios heighten intra-sexual competition among men, we focus on male behaviors and the norms that regulate them: masculinity norms. These norms can be defined as the culturally accepted rules and standards that guide and constrain men’s behavior within society. To measure how much men adhere to such norms, Mahalik et al. (2003) developed the Conformity to Masculinity Norms Inventory (CMNI).

The CMNI is a multi-dimensional scale that measures to what extent an individual man’s actions, thoughts, and feelings conform to the dominant masculinity norms in Western societies. It captures 11 distinct masculinity norms: winning; emotional control; risk-taking; violence; dominance; playboy; self-reliance; primacy of work; power over women; disdain for homosexuals; and pursuit of status. We hypothesize that skewed sex ratios can influence masculinity norms that, once ingrained in local culture, continue to manifest themselves in present-day behaviors.^13^ Such cultural persistence can be explained by both hysteresis due to parental transmission (Bisin & Verdier, 2001) and by conformity and peer effects (Acemoglu & Jackson, 2017; Ushchev & Zenou, 2020).

Based on the CMNI framework, we expect that areas that were historically characterized by male-biased sex ratios and, therefore, intense male-male competition, developed stricter masculinity norms that continue to manifest themselves across four broad domains: (i) violence and bullying; (ii) risk taking, help avoidance and unhealthy behavior; (iii) male-stereotypical occupational segregation; and (iv) negative attitudes towards homosexuals.

The underlying mechanism of interest is the intensification of male-male competition generated by male-biased sex ratios. As explained in the Introduction, we therefore focus on behaviors for which inter-sexual cooperation would predict behaviors that are either opposite or unrelated to the ones generated by intra-sexual competition, such as cooperation versus violence. We now explain in more detail how sex ratios likely influence behaviors in our four domains of interest.

First, in line with an effect of skewed sex ratios on violence and aggression, studies have documented that unmarried men—those exposed to intense competition for access to females—are more likely to commit crimes, including rape, murder, and assault (Sampson et al., 2006; Henrich et al., 2012). Accordingly, we examine outcomes such as violent assault, sexual offenses, as well as bullying in schools. Bullying in schools should also be understood as capturing the socialization process through which masculinity norms are imposed and transmitted to younger generations. Peers at school are a major influence on the development of gender normative behavior in childhood and adolescence (Adler et al., 1992; Leaper & Farkas, 2014).

Second, intense male-male competition is expected to favor risk taking, self-reliance and help avoidance, which may lead to increased morbidity and earlier death. As a proxy for risk taking, and a measure of intermediary outcomes that helps to ‘decompress history’, we use voluntary recruitment in WWI. Appeals to masculinity, including by public shaming of men not wearing uniforms as cowards, were a key driver of volunteering in WWI, especially in Australia where all recruits were volunteers (Becker, 2021; Inwood et al., 2020).

Men adhering to hegemonic masculinity norms attach a stronger stigma to mental health problems, are more likely to avoid health services (Good et al., 1989; Latalova et al., 2014) and are more likely to think about suicide (Pirkis et al., 2017). As a proxy for the avoidance of preventative health care we use answers to a survey question we commissioned about help-seeking behavior, a survey question about prostate cancer screening, as well as as deaths by suicide and prostate cancer rates. Prostate cancer is often curable if treated early, but avoidance of diagnosis and treatment is a major public health concern. A large medical literature has established a clear relationship between adherence to a masculine identity and the avoidance of prostate cancer screening.^14^

A third manifestation of male identity for which we test, is occupational choice. The role of identity in determining job choice has been discussed since Akerlof and Kranton (2000). More recently, the role of masculine identity in preventing men from taking up occupations that are perceived as stereotypically female has attracted attention as a driver of so-called retrospective wait unemployment (Katz, 2014) and of occupational sorting between stereotypically male and female jobs (that is, occupational gender segregation). Milner et al. (2018) show for Australia that men in male-dominated jobs report greater adherence to masculine norms.

Fourth, the effect of higher historical sex ratios (and male-male competition) on attitudes towards homosexuality is a priori ambiguous. Male homosexuality should, at first sight, be welcomed, as it reduces the number of male competitors for scarce women. However, as explained above, the primary effect of a male-biased sex ratio is to intensify male-male competition. In their strife for dominance, men will aim to (often publicly) subdue other men, in particular those who do not display strong markers of masculinity and are perceived as easier targets, thereby encouraging bullying and aggression towards males perceived as not masculine enough (Franklin, 2000; Parrott & Zeichner, 2008; Vincent et al., 2011).

Men display sexual prejudice both to establish and reaffirm their own masculinity and to punish other men who fail to meet gender role requirements (Herek & McLemore, 2013). Indeed, the dread of being perceived as gay and the primacy of being thought to be heterosexual are among the strongest components of the CMNI scale, and correlate positively and significantly with other dimensions of masculinity, such as dominance, risk-taking, an inclination for violence, and negatively with emotional openness and help-seeking behavior. We will proxy this masculinity norm by opposition against same-sex marriage, which we measure using voting records from the 2017 nation-wide referendum on same-sex marriage and the results of a large-scale household survey.^15^

To sum up, we expect that historically male-biased sex ratios led to heightened norms of masculinity as expressed in violent behavior and bullying; help avoidance and unhealthy behavior; occupational gender segregation; and less support for same-sex marriage.

## Historical background

### The arrival and allocation of convicts

Between 1787 and 1868, 132,308 male and 24,960 female convicts were transported from Britain to Australia. The 1836 and 1842 censuses in New South Wales and Tasmania showed that the average convict sex ratio stood at more than 28 men for every woman (Table 1). These convicts, who constituted the founder settler population of Australia, were far from being hardened criminals guilty of violent crime. Instead, they were quite representative of the Victorian working class at the time in terms of, for example, their occupations, literacy, numeracy, and height (Nicholas, 1988; Oxley, 1996; Meinzer, 2015). Based on evidence on violence-related injuries such as fractures, scars, and cuts, Meinzer (2015) concludes that convicts were not especially prone to violence as compared with the general population in Great Britain. Indeed, two thirds of transported convicts were first offenders of minor property crime, such as petty theft (Nicholas, 1988).^16^

**Table 1:** Sample characteristics and balance

	Mean	SD	Coefficient on Convict SR (standardized)	*p*-value	Obs
	(1)	(2)	(3)	(4)	(5)
*Panel A: Historical data (county level) & Geographic features (postcode level)*					
Convict sex ratio	28.39	42.4			34
Historical sex ratio	3.84	2.5	0.38	0.00***	34
Historical population (1000 s)	3.45	6.6	0.12	0.21	34
Number of convicts (1000 s)	0.98	1.5	–0.09	0.34	34
Share employed in agriculture	0.24	0.1	0.08	0.38	31
Share employed in domestic service	0.17	0.2	0.04	0.64	31
Share employed in manufactoring/mining	0.14	0.2	–0.05	0.61	31
Minerals: none	0.19	0.4	–0.10	0.10	515
Minerals: coal	0.54	0.5	0.06	0.34	515
Minerals: gold	0.25	0.4	0.08	0.32	515
Landforms: plains, plateaus	0.19	0.4	–0.10	0.10	515
Landforms: mountains	0.79	0.4	0.05	0.40	515
Mean annual rainfall in 1901	219.25	21.3	–0.04	0.65	515
Soil: toxicity	0.91	0.3	–0.01	0.86	515
Soil: excess salts	0.97	0.4	–0.01	0.57	515
Soil: oxygen availability to roots	1.02	0.4	0.04	0.31	515
Soil: nutrient retention capacity	1.37	0.7	–0.03	0.47	515
Soil: nutrient availability	1.60	0.9	–0.03	0.44	515
Mapped water bodies (% postcode)	4.87	8.4	–0.04	0.25	515
*Panel B: 2011/2016 Census (SA1 level controls)*					
Contemporary population (100 s)	4.20	1.8	–0.03	0.24	16,611
Contemporary sex ratio	1.03	0.5	–0.01	0.22	16,611
Urban	0.96	0.2	–0.08	0.44	16,611
% Under 30 years old	0.39	0.1	–0.04	0.61	16,611
% Foreign born	0.28	0.2	–0.25	0.08*	16,611
Unemployment rate (male)	0.06	0.0	–0.06	0.30	16,588
Unemployment rate (female)	0.06	0.0	–0.09	0.12	16,588
% Completed high school (year 12)	0.42	0.1	–0.05	0.81	16,611
Median HH weekly income	1606.11	637.8	0.02	0.89	16,611
Buddhist	0.03	0.1	–0.12	0.08*	16,611
Anglican	0.17	0.1	0.14	0.43	16,611
Catholic	0.26	0.1	–0.07	0.19	16,611
Other Christian	0.15	0.1	–0.05	0.33	16,611
Muslim	0.03	0.1	–0.13	0.05**	16,611
No religion	0.23	0.1	0.16	0.13	16,611
*Panel C: HILDA survey on attitudes and norms (individual-level controls)*					
Age	45.15	18.8	0.01	0.67	8,838
Male	0.46	0.5	0.01	0.01**	8,838
Australia-born	0.75	0.4	0.06	0.42	8,838
Beyond year 12 education (male)	0.62	0.5	0.01	0.75	4,107
Beyond year 12 education (female)	0.55	0.5	0.01	0.90	4,731
Income (log, male)	11.28	0.9	0.02	0.62	4,105
Income (log, female)	11.20	0.9	0.05	0.42	4,730

Column (3) contains the coefficient from a regression of the variable listed in the first column on Convict Sex Ratio (CSR), with both variables standardized such that the coefficient is interpreted as the change (in standard deviations) due to a one standard deviation increase in the CSR. Column (4) provides the *p*-value from the test of whether the coefficient in column (3) is equal to zero. Column (5) contains the number of observations for which we have data at the level the data are reported (historical counties, postcodes, SA1s, or individual-level). All data that is not individual-level is matched to SA1s (the smallest statistical geographical unit) for use in regressions

*$$p<$$ 0.1, **$$p<$$ 0.05, ***$$p<$$ 0.01

**Fig. 1:**
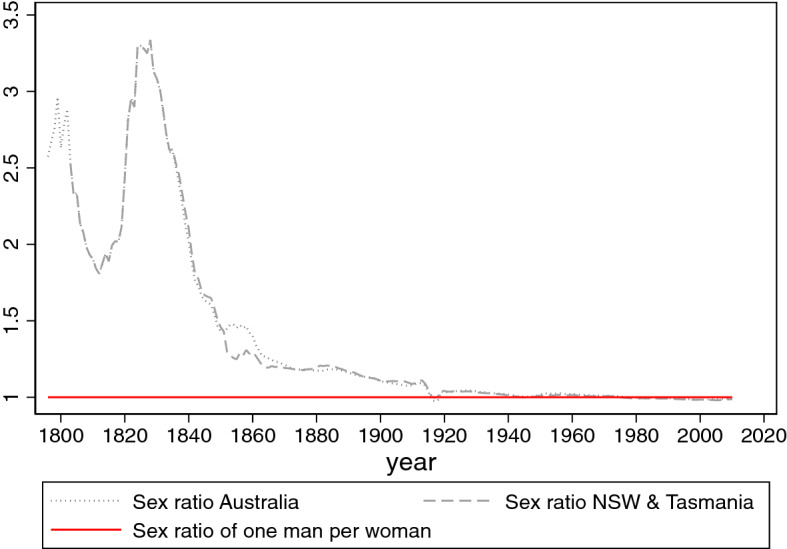
The sex ratio in Australia: Number of men to every woman, 1788–2011 *Source:* Australian Bureau of Statistics

Once in Australia, convicts were not confined to prisons but were assigned to work, first under government supervision and later, as the number of free settlers and emancipists (ex-convicts) grew, under the direction of private employers. They were generally freed after the term of their sentence, usually seven years. Convicts made up as much as 38% of the population in the colonial Censuses of New South Wales and Tasmania that we use in this study.^17^ Voluntary migration was very limited and mainly involved men migrating in response to male-biased economic opportunities available in agriculture and, after the discovery of gold in the 1850s, mining. Because of the predominance of male convicts and migrants, male-biased population sex ratios endured in Australia for more than a century, although less severely after the end of convict transportation (Fig. 1).

Using the sex ratio among convicts alleviates the self-selection issue that free men and women chose their location based on unobservable preferences. Convicts were not free to choose where to live but were allocated centrally on the basis of local labor needs. As part of our identification strategy, which we describe in more detail in Sect. 5, we therefore condition on a comprehensive set of proxies for local economic opportunities at the time. Identification then rests on the assumption that the spatial distribution of the relative number of convict men and women was as good as random once we control for historical employment sector shares and for geographic factors, including the location of minerals and land type.

Historical and cliometric evidence supports the idea that convicts were assigned on the basis of local labor requirements, which we can control for. One might worry that local convict populations differed not only in terms of their sex ratio but also in terms of other characteristics that may transmit across generations. For example, especially violent men may have been sent to (remote) areas with more male-biased sex ratios. Our results might then not only reflect the lasting impact of skewed sex ratios per se but also spatial variation in violent tendencies among men (which may have transmitted genetically or behaviorally over time).

There exists, however, little to no historical evidence supporting such an interpretation. Meredith (1988) describes how convicts were assigned according to their abilities and not ‘with reference to their sentence, crime or general ‘character”. As described by Governor Bligh of New South Wales in 1812: “*They (the convicts) were arranged in our book for the purpose of distinguishing their ages, trades, and qualifications and whether sickly, or not, in order to enable me to distribute them according*” (Meredith, 1988, p. 15, emphasis added). The treatment and assignment of a convict ‘*bore no relation to his crime, general character and behavior or the length of his sentence*’ (ibid, p. 19). According to Bligh: ‘*If one person convicted of a great offense, and another of an inferior one, come out together, the Governor, having no such information, is not enabled to distribute them in reference to that circumstance; upon their arrival in the settlement they are all treated alike*’ (ibid, p. 19). A convict’s previous crime and character were ‘points that are altogether overlooked’ and spatial allocation happened ‘not upon any retrospect of their former lives, or characters, or the length of their sentencing’. The Select Committee on Transportation concluded in 1837 that ‘Therefore on the whole, it must be a mere lottery with regard to the condition of the convict’ (Meredith, 1988, p. 20).

### Masculinity norms in Australia: historical context

As a nation of convicts transported from Great Britain, perceptions of manhood in colonial Australia initially developed from those prevalent in Britain at the time. Tosh (2005) describes how this masculine identity was shaped by the urbanization and industrialization of England: Men were increasingly seen as a household’s main breadwinner and judged by their physical strength and courage. Brady (2005, p.22) describes these Victorian masculinity norms as hegemonic, in the sense that they “*emphasised the authority of the paterfamilias over his wife and family, but also stigmatised masculine traits that undermined his position*.”

Any traits and behavior that were not in line with the prevailing norms, such as same-sex sexual activity or effeminate behavior, were considered to undermine the patriarchical system. Indeed, this was exemplified by the strong condemnation of sexual encounters among male convicts by Australia’s colonial authorities. The authorities perceived homosexual activity to be widespread among convicts and worried that “*In future years a moral stain of the deepest dye may be impressed, perhaps immovably, on its people, and thus become attached to the name of the Englishmen”*, according to Norfolk convict department magistrate Pringle Stuart quoted in Hughes (2003, p.271).^18^

The masculinity norms imported from Britain became rapidly interwoven with an emerging masculine identity that was shaped by the harsh reality of bush life for men without female companionship. Russel Ward’s influential book of Australian history traces the origins of the Australian legend or national *mystique* (Ward, 1958). This mystique, which is largely masculine in nature, is closely intertwined with the quintessential component of Australian male heritage called ‘mateship’. Mateship not only embodies equality, loyalty, and friendship between men (Pease, 2019) but also “*a lack of emotional expression other than sharing jokes”* (Edgar, 1997; p.79). Other features of this emerging Australian masculinity included heavy drinking, gambling, and, more recently, homophobia (Pease, 2019).^19^

Ward (1958) argues that mateship and the emerging Australian masculine identity not only reflected the material conditions of pastoral life in the outback but also the fact that “*the first and most influential bush-workers were convicts or ex-convicts, the conditions of whose lives were such that they brought with them to the bush the same, or very similar attitudes”* (p.2). These masculinity norms became pervasive and persisted because “*[t]he pastoral industry was, and still is, the country’s stable. Its nature, the nature of Australian geography, and the great though decreasing scarcity of white women in the outback, brought into being an itinerant rural proletariat, overwhelmingly masculine in composition and outlook.*” (p.10). The emerging Australian masculine identity therefore effectively fused generic British masculinity norms with local values that reflected bush life and, in particular, the absence of female companions. It is this latter component that varied so starkly across Australia’s vast territory and that imprinted masculinity norms of different strength in different parts of the country.

Importantly, these spatially variegated masculinity norms then became further entrenched in the First World War, with the Anzac (Australian and New Zealand Army Corps) as the leading exemplar of idealized masculinity. In particular, “*According to the legend, the ‘heroic Anzac’ takes over from the ‘noble bushman’ as the embodiment of the ‘typical Australian’. The frontier masculinity is merged with that of the warrior, and mateship is re-configured for the trenches”* (Murrie, 1998). In the patriotic rhetoric of the day, anyone who was not an Anzac, especially an eligible man who had chosen not to volunteer, could not claim the same manliness” (Dwyer, 1997).

## Data

We combine various data sets on historical and modern-day Australia by matching the earliest possible historical Census in each state to: (i) WWI veterans; (ii) modern-day postcode- or local government area-level data on violence, crime, and mortality; (iii) modern-day nationally representative surveys of attitudes (HILDA) and of the lives and experiences of children (LSAC); (iv) present-day Census data on occupations; and (v) data on the 2017 referendum on same-sex marriage. The “online Appendix” provides a comprehensive list of all data sources and variable definitions.

### Historical data

#### Convict sex ratios and balance tests

Our measure of the historical convict sex ratio comes from the first reliable Census in each state, as available from the Historical Census and Colonial Data Archive. We focus on the earliest possible Census in a state to measure convict population before the onset of mass migration, when convict shares of the population were highest. Although the population of Australia at the time was only about 255,000 people, 29% of the current population of Australia lives in areas covered by these historical data.

Only New South Wales (which included at the time what is now the Australian Capital Territory) and Tasmania were penal colonies. We use the 1836 New South Wales Census^20^ and the 1842 Tasmanian Census.^21^ The unit of observation in the Census is a county.^22^ 34 counties harbored convicts. The average county had 3,446 individuals and most counties (about 95%) had between 300 and 10,000 people. The historical Censuses also contain data on economic occupations.

Table 1 displays descriptive statistics and shows how covariates are balanced by regressing each characteristic on the (standardized) convict sex ratio. Agriculture was the largest employment sector in Australia at the time, accounting for 24 percent of the labor force. Domestic services followed at 17 percent, and then manufacturing and mining with a combined total of 14%. The shares of people employed in these major sectors historically are not statistically related to the convict sex ratio (see Panel A of Table 1). Still, we control throughout our analysis for the historical shares of employment in different sectors, which may have influenced where colonial administrators assigned convicts.

**Fig. 2:**
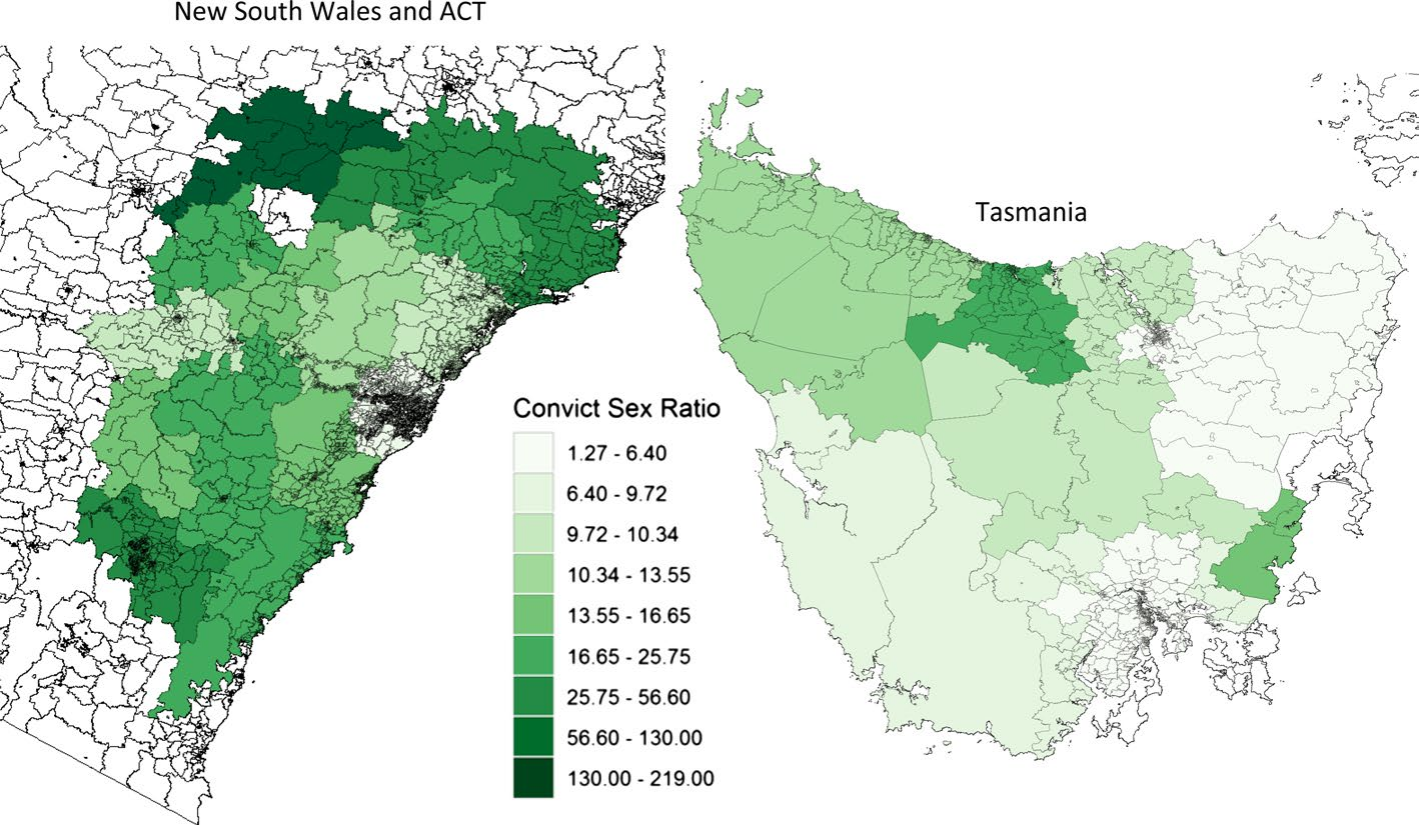
Convict sex ratios in mid-19th century Australia. *Notes:* The maps show the parts of Australia that had convict settlement: Australian Capital Territory, New South Wales, and Tasmania. Boundaries depicted are for the 2016 Statistical Areas Level 1 (SA1), the smallest unit for the release of census data. *Source*: Australian Historical Censuses and Volume 1 of the Australian Statistical Geography Standard

For the same reason, we also control for land characteristics and mineral endowments, as counties with a high convict sex ratio tended to have more gold deposits and more rugged terrain. Figure 2 maps the convict sex ratio across New South Wales and Tasmania. The concentration of convicts of both sexes does not have a definite pattern: high and low sex ratios were found in the hinterland as well as along the coast.

#### Voluntary service in WWI

All recruits for WWI military service were volunteers. We use data from the 1933 Census on veterans who served in WWI as a proxy for voluntary enlistment in the first World War. 5.8% of men and 0.04% of women in 1933 report service in WWI. Digitized data on WWI enlistment linked to places of origin of volunteers is only available for Tasmania from Inwood et al. (2020). Using the presence of veterans in 1933 as a proxy for voluntary enlistment may suffer from measurement error due to survival bias, reporting bias, and post-war migration, which would be problematic if correlated with the convict sex ratio.

To gauge the extent of measurement error and the potential for it to be correlated with the convict sex ratio, we compare enlisted individuals and surviving veterans in 1933 at the level of 52 municipalities in Tasmania for which we have both sources of data. A comparison of the number of volunteers with the number of veterans implies a death rate of 18.3% between enlistment and 1933. This is very close to the actual military fatality rate, estimated for Tasmanian troops in WWI at 19.2% (Inwood et al., 2020). The correlation between enlisted volunteers and WWI veterans in 1933 is very high, at 0.95, suggesting limited measurement error. Combat rotation in WWI was organized at the level of brigades and battalions, which themselves were structured on a state basis.^23^ State fixed effects would thus capture the main driver of fatality (that is, combat rotation to specific battles on specific days) limiting concerns about systematic correlation between fatality rates and local sex ratios.

We still check that the local difference between the number of veterans in 1933 and the number of men enlisted in WWI is uncorrelated with the convict sex ratio. The raw correlation coefficient (0.07) is small and statistically insignificant, suggesting limited roles played by selective fatality, misreporting, or migration.^24^

### Data on present-day outcomes

To explore the long-run effects of male-biased sex ratios, we use several data sources. First, we obtain crime statistics at the postcode level from the police or statistical agencies in respective states. As described in Sect. A.5 of the “online Appendix”, crime reporting varies across states but we are able to build consistent categories of crime between 2006 and 2016. We match these data to the 2006, 2011, and 2016 Census and interpolate the population between Census years to compute crime rates per capita.

Second, we use mortality statistics to obtain rates of death attributable to suicide and other forms of preventable mortality due to help avoidance. Data is from the Mortality over Regions and Time 2011–2015 data set (Australian Institute of Health and Welfare). The dataset lists the top 20 causes of death by gender and local government area (LGA) over this time period, as well as the total number of deaths in each year. Our main proxies for help avoidance behavior consist of mortality from prostate cancer and suicide. Moreover, a nationally representative survey, HILDA, gives us access to detailed and representative data on whether male respondents (aged 50+) had a prostate examination in the past twelve months.

Third, we use data from the 2011 and 2016 Census on the share of men and women across all 4-digit occupations. We first classify occupations into three groups: feminine, masculine, or neutral. To ensure that we pick up occupations that are known to be “stereotypically male/female”, we classify the most common occupations at the 4-digit level (occupations with total employment shares greater than 0.5%, approximately 55 of a total of 469 occupations, with 55% of the workforce represented in these occupations).

These common occupations are then considered feminine, neutral, or masculine if their national male share in the occupation is less than 33% (feminine), between 33 and 66% (neutral), or over 66% (masculine). Examples of the most masculine occupations are ‘Carpenters and Joiners’, ‘Metal Fitters and Machinists’, and ‘Motor Mechanics’ (all 99% male). Examples of the most feminine occupations are ‘Child carers’ (4.9% male), ‘Receptionists’ (5.2%), and ‘Education Aides’ (9.6%). Neutral occupations include ‘Real estate sale agents’ (50.0% male) and ‘Retail managers’ (50.5%).

Fourth, to measure the extent to which historical sex ratios have shaped attitudes towards homosexuals, we use the results of the 2017 referendum on same-sex marriage. The Australian Marriage Law Postal Survey was conducted by the Australian Bureau of Statistics (ABS) as a postal vote. Unlike electoral voting, which is compulsory in Australia, responding to the survey was voluntary. A survey form was mailed to everyone on the electoral roll, asking the question *"Should the law be changed to allow same-sex couples to marry?"*. Data is available at the level of 150 electoral districts. The results showed that 61.6% voted in favor of marriage equality across the country while 38.4% voted against it. Turnout was high, at 79.5%. While the postal survey was non-binding, the Liberal–National Coalition government had pledged to support a Parliamentary bill to legalize same-sex marriage in case of a *"Yes"* outcome. A few weeks after the vote, Australia’s House of Representatives voted in favor of legalizing same-sex marriage.

The district-level postal vote data provide us with a clean manifestation of masculinity norms, as negative attitudes towards sexual minorities are at the heart of such norms (Mahalik et al., 2003). The vote data are also unique in that they provide us with an ‘undiluted’ measure of people’s support for a salient normative cause (electoral voting would conflate these issues with many others, including economic considerations). Moreover, anonymous voting is not susceptible to response bias that can plague surveys. However, this data does not allow for individual comparisons.

To exploit individual variation, we also use HILDA, which identifies respondents through their residential postcode and contains a wide range of socio-demographic individual characteristics. Of interest is the question on attitudes towards the enfranchisement of sexual minorities: *"Homosexual couples should have the same rights as heterosexual couples do"*. Answers range from 1 (strongly disagree) to 7 (strongly agree), and we categorize individuals as broadly supportive of same-sex rights if they answered 4 (neutral) or above.

Fifth, we use survey measures of norms regarding help avoidance. We use data from Taking the Pulse of the Nation (TTPN), a repeated cross-sectional survey of 1200 adults carried out every two weeks between October 2020 and December 2021 about experiences with COVID-19. We use a question about willingness to get inoculated with a COVID-19 vaccine.^25^ Masculinity norms have been highlighted as an obstacle to preventative measures against the spread of COVID-19: Men are less likely to wear a face covering than women and are more likely to associate wearing a covering with “weakness” (Capraro & Barcelo, 2020).

We also commissioned the Melbourne Institute to include the CMNI question that best predicts the outcomes that we study (i.e. violence, intimate partner violence, suicide attempts, doctor visits, see Table 8) in the latest HILDA survey round. This question asks on a five-point Likert scale whether the statement “*It bothers me to ask for help*" describes the respondent.

Lastly, to refine our understanding of possible socialization mechanisms that sustain the relationship between historical sex ratios and modern-day male identity and behavior, we use data on bullying in schools from a nationally representative survey of Australian youth (LSAC). LSAC is a longitudinal study of 10, 000 children, now teenagers, since 2003. It follows two cohorts (aged 0–1 in 2003–2004, and 4–5 in 2003–2004) and examines a broad range of questions on development and well-being. In particular, the survey measures the incidence of child bullying at school as reported by parents, children, and teachers. Due to a large number of missing observations from children’s reports we focus on responses by parents and teachers.

As explained before, we choose these outcomes as behavioral manifestations of norms of masculinity that are unrelated to male–female bargaining, or that even operate in domains in which the effect of male–female bargaining should go in the opposite direction as the effects of male–male competition. Our leading example is violence: we expect within-sex competition to select for violence as a mean of establishing oneself in the male hierarchy, while women would instead select cooperative men and turn away from violent men (who can be dangerous for themselves and for their children).

**Fig. 3:**
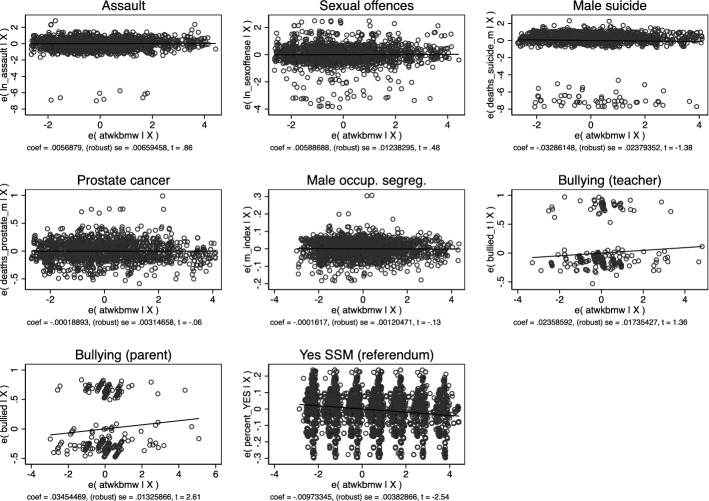
Partial correlations between masculinity norms and gender-roles norms. *Notes:* Partial correlations between each proxy of masculinity norms (as indicated in the graph header) and attitudes towards gender roles. The measure of attitudes towards gender roles is the same as the one used in GK: question *atwkbmw* in HILDA, which asks respondents to what extent they agree with the statement: “It is better for everyone involved if the man earns the money and the woman takes care of the home and children”. Response categories range from 1 (strongly disagree) to 7 (strongly agree) (mean value in the sample with historical information: 3.33 (s.d.: 1.91)). The set of controls corresponds to the specifications reported in the paper (without controlling for the convict sex ratio) (see controls included in Tables 2, 3, 4, 5 and 7 for each respective outcome)

To examine this more formally, we calculate the correlation between these proxies for masculinity norms and proxies for gender norms that reflect male–female bargaining. To measure the latter, we focus on a HILDA survey question that GK use as a key proxy for the strength of gender-role norms influenced by male–female bargaining: the extent to which respondents agree that “*It is better for everyone involved if the man earns the money and the woman takes care of the home and children*”. As shown in Fig. 3 the proxies for masculinity norms that we use in this paper are largely uncorrelated with attitudes towards gender roles.^26^

### Data matching

To match present-day to historical data, we project all our data on the smallest geographic unit in the Census (SA1). We rely on the historical boundaries established by GK, which we project again at the SA1 level (as opposed to the larger postcode level used in GK). We then match all our outcome data to the 2011 or 2016 Census at the SA1 level and to the historical data. We match the 1933 Census data at the level of the smallest geographic area for which data is available, the local government area (LGA).

We retain the following SA1 characteristics from the Census as possible controls: present-day sex ratio, population, urbanization, religious composition, unemployment (by gender), education, age, and percentage Australian born. Across all specifications, controls are consistently measured at the SA1 level. We also collect data on minerals, soil quality, and land type from Geoscience Australia. Table 1 provides descriptive statistics. We present the balance of covariates in columns 3-4. There are no statistically significant differences of meaningful size across high versus low convict sex ratio areas in terms of present-day sex ratio, urbanization, age, male or female unemployment, income, or education. Of the 39 balance tests conducted in Table 1, four are statistically significant at the 10% level (of which two at the 5%), consistent with what we should expect to happen by chance.

## Empirical strategy

We examine the long-term effects of male-biased sex ratios on present-day outcomes by estimating the following equation:

5.1 $$\begin{aligned} y_{ijcs} = \alpha + \beta CSR_{cs} + X_{jcs}^{G\prime }\Gamma +X_{cs}^{H\prime }\Pi + T_{jcs}^{C\prime }\Lambda + X_{ijcs}^{C\prime }\Theta + \delta _s + \varepsilon _{ijcs} \end{aligned}$$

Where $y_{ijcs}$ are outcomes for individual *i* in modern statistical area *j* (SA1, postcode, or LGA), part of historical county *c*, in state *s*. $CSR_{cs}$ is the historical convict sex ratio: the number of male convicts to female convicts in historical county *c* in state *s*. We transform this variable into a z-score so that we can interpret the estimated coefficients as the impact of a one standard deviation increase in the historical convict sex ratio. $\delta _s$ is a vector of state dummies. Outcomes are measured at the individual level, SA1 level, postcode, or LGA depending on data availability.

Since historical data at the level of historical counties is less granular than present-day data at the SA1 or individual level, we cluster standard errors at the historical county level. As only New South Wales and Tasmania were penal colonies, convicts were present in 34 historical counties. In “Appendix Table 9”, we correct our main estimates with the wild cluster bootstrap method based on 1,000 replications, following Cameron et al. (2008), to account for the limited number of clusters. We also consider the possibility that our results might (partially) reflect spatial autocorrelation in the residuals (Kelly, 2019). We present in “Appendix Table 9” Moran statistics that mitigate concerns that our results merely reflect spatial noise. Moreover, throughout all tables we report standard errors that are spatially heteroskedasticity and autocorrelation consistent (HAC-robust, cf. Conley (2010)).

$X_{jcs}^G$ and $X_{cs}^H$ are vectors of time-invariant geographic and historic characteristics that may correlate with the convict sex ratio and might still influence present-day outcomes. The need to develop the colony of Australia, chiefly in agriculture and mining, may have influenced where convicts were assigned. This could bias our estimates if initial economic specialization persisted over time and influences our outcomes of interest through its lasting influence on present-day conditions.

To flexibly account for geographic differences that may correlate with agricultural potential, we control for latitude and longitude of each postcode’s centroid. To control more precisely for mining and agricultural opportunities, we control for mineral deposits and land characteristics. We also control for county historical economic specialization by including in $X_{cs}^H$ the shares of the population employed in the main categories of employment in 19th century Australia: agriculture, domestic services, mining and manufacturing, government, and learned professions. Total historical population in the county is also included in $X_{cs}^H$.

$T_{jcs}^C$ and $X_{ijcs}^C$ are vectors of SA1-level and individual-level present-day controls. The full baseline controls account for 37% of the raw variation in the convict ratio, leaving 67% for identification (see also “Appendix Fig. 4”). Although present-day sex ratios and urbanization are uncorrelated with the historical convict sex ratio (Panel B of Table 1), these factors are important drivers of attitudes towards sexual minorities (Stephan & McMullin, 1982) and crime (Glaeser & Sacerdote, 1999). For this reason, we include controls for present-day sex ratio, population, and urbanization at the SA1 level.^27^

A related concern is the potential influence of religion. Historically, there was little variation across counties in religious affiliation, with the main groups being evenly distributed across areas. In the 1836 New South Wales Census, 67% of the population was Protestant and 33% was Catholic, with a standard deviation of 0.13 for the two distributions across counties, and no statistically significant difference across high and low convict sex ratio areas. Today, we observe no statistically significant differences in the shares of main religions across high versus low convict sex ratio areas, (Panel B of Table 1), although the share of people who identify as Muslim is slightly lower in areas that had higher convict sex ratios. Still, because of the potentially large influence of religiosity on risk-taking, violent behavior and attitudes towards same-sex marriage, we will include the shares of religious groups at the SA1 level as additional controls in robustness tests (Sect. 7.1).

In the models using individual survey data, individual controls are gender, age, and whether the respondent was born in Australia. These characteristics do not vary systematically with the historical convict sex ratio (Panel C of Table 1). Present-day sex ratio, urbanization, unemployment for men or women, income, education, and age are also uncorrelated with the convict sex ratio (Panel B of Table 1, based on the Census). This suggests that the convict sex ratio was not systematically related to other characteristics that may influence present-day outcomes.

To identify a causal effect of the historical convict sex ratio in Eq. 5.1, we need to assume that the spatial distribution of the relative number of convict men and women was random, conditional on our proxies for economic opportunities and total population at the time. Convicts were not free to choose where to live, and were allocated centrally on the basis of observable characteristics. Using the sex ratio among convicts therefore alleviates the self-selection issue that free men and women chose their location based on unobservable preferences. That said, as discussed in the historical background section, convict assignment was not purely random but may also have been influenced by labor requirements. We remove this potential endogeneity bias by controlling for historical employment sector shares and for geographic factors, including the location of minerals and land type.

We choose to report reduced form estimates based on the sex ratio among convicts, rather than use the convict sex ratio as an instrumental variable for the historical population sex ratio, for two reasons.^28^ First, our suggested mechanism is that the sex ratio shapes attitudes through its effect on mating competition. It should therefore only operate through the sex ratio among adults of reproductive age (ASR). However, the historical Censuses do not systematically break down the population by age, and many individual records have been destroyed, so that we cannot compute the ASR. The population sex ratio is thus a noisy measure of the treatment of interest. Convicts were generally of marriageable age, so that the sex ratio among convicts is a more precise proxy of an ASR. Second, while the convict sex ratio and the population sex ratio are highly correlated ($\rho$ = 0.72) and our results are robust to an instrumental variable specification (Table 15), we believe the reduced form approach is statistically more appropriate given the sample size (Lee et al., 2020; Young, 2020).

Causal identification also requires that the convict sex ratio only influenced present-day outcomes through its effect on male-male competition. We have already discussed that male-biased sex ratios also influence male–female bargaining. However, as explained, the effects of sex ratios that are channeled through male–female bargaining are expected to, if anything, dampen our effects, causing us to underestimate the pure effect on male-male competition.

The presence of convicts itself may also have had a direct effect on health, crime and electoral outcomes today. Furthermore, it is possible that more hardened, risk-loving and violent convicts were systematically sent to more male-biased areas. Such endogenous selection could generate a correlation between, on the one hand, the convict sex ratio and, on the other hand, preferences for risk and violence stemming from convictism itself, which may have persisted until today. Historical evidence reduces this concern. First, as we describe in Sect. 3, convicts that were deported to Australia were not hardened criminals guilty of violent crime. Instead, they were mostly first-time offenders of petty property crime. Second, the placement of convicts was decided in a highly centralized way, making it unlikely that the spatial distribution was determined by unobservable taste for risk. Nevertheless, throughout all specifications we control for the number of convicts, together with total historical population. This absorbs the legacy of convictism as separate from the legacy of the sex ratio. To address the possibility that the relationship between the number of convicts and the sex ratio among convicts was not mean preserving, that is: only the more hardened, risk-loving and violent *male* convicts were systematically sent to more male-biased areas, we also perform our analysis with the total number of *male* convicts rather than the overall convict population.^29^

## Empirical results

This section first discusses the medium and long-term consequences of male-biased sex ratios on violence and crime; health; and occupational gender segregation. We then provide evidence from the 2017 same-sex marriage referendum.

### Risk-taking: voluntary service in WWI

**Fig. 5:**
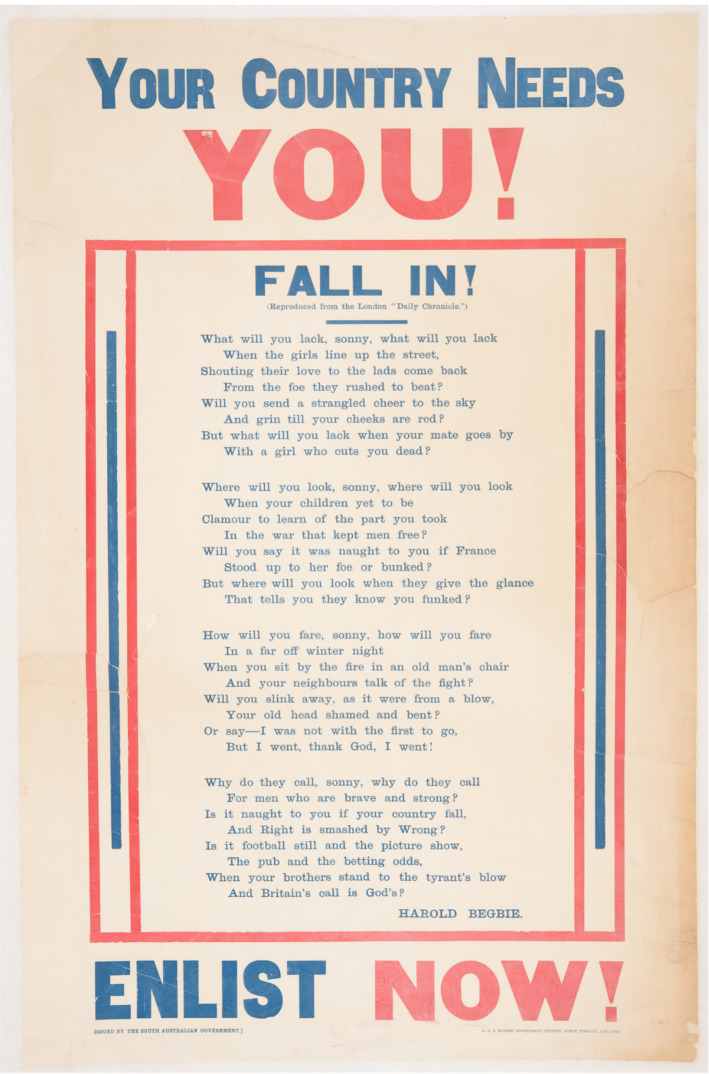
Example of a WWI recruitment poster in Australia. *Notes:* Example of a WWI recruitment poster in Australia. Source: Public Library, Museum and Art Gallery of South Australia, State Records’ series GRG32/16

Military service in WWI was a risky endeavor. Fatality rates are estimated at 19.2% among Tasmanian recruits (Inwood et al., 2020), higher than those in the French military (estimated at around 16% (Gay, 2021)). Appeals to masculinity and the public shaming of unenlisted young men for their cowardice were key drivers of enlistment (Becker (2021), see also Fig. 5). We therefore expect a positive relationship between historical sex ratios and voluntary recruitment. We test this hypothesis and report the results in column 1 of Table 2. The estimates show that the rate of voluntary recruitment in WWI among men was significantly higher where the convict sex ratio had been more skewed. The point estimate indicates that a one standard deviation increase in the convict sex ratio is associated with a 5.6% increase in the share of men who volunteered to serve in WWI.

**Table 2:** Historical convict sex ratios and violence

	WWI participation (1933 Census)	Present-day violence	
	Percent of men who served	Assault ln (Incidence per 100K)	Sexual offenses ln (Incidence per 100K)
	(1)	(2)	(3)
Convict sex ratio (z)	0.323**	0.088**	0.128**
	(0.132)	(0.036)	(0.053)
Spatial HAC *p*-value	0.002	0.005	0.017
Observations	162	16,578	16,578
$R^{2}$	0.42	0.26	0.59
Mean of dependent var	5.79	834.00	125.14
Number of clusters	34	34	34
State FE	Yes	Yes	Yes
Geographic controls	Yes	Yes	Yes
Historical controls	Yes	Yes	Yes
Minerals and land type	Yes	Yes	Yes
Contemporaneous SR and pop	Yes	Yes	Yes

Standard errors clustered at the historical county level. ‘Geographic controls’ are at the postcode level and include the postcodes centroid and the minerals and land type of the postcode. ‘Minerals and land type’ is the presence and type of mineral deposit (major coal; major gold; other) and land formation (plains and plateaus, mountains, other), which are provided by Geoscience Australia. ‘Historic controls’ are: the historical county population, convict population, as well as the proportion of residents working historically in agriculture, domestic service, manufacturing and mining, and government services and learned professions. ‘Present-day SR and population’ are the number of men to women (SR) at the postcode, the total population density of the SA1, whether it is urban, and its population. Demographic data are averages from the 2011 and 2016 Census. Data for Column 1 comes from the 1933 Census at the LGA level. ‘Contemporaneous SR and population’ are the number of men to women (SR) at the postcode (present-day) or LGA (1933) and its population. In Columns 2–3, the mean of the dependent variable is reported as the untransformed rate of incidents per 100,000

*$$p<$$ 0.1, **$$p<$$ 0.05, ***$$p<$$ 0.01

### Violence, suicide, and health

We investigate the long-term consequences of male-biased sex ratios on violence in columns 2 and 3 of Table 2. Crime data are reported at the postcode level, which we project to the SA1 level. The dependent variables are the natural logarithm of the mean number of assaults and sexual offenses per 100,000 inhabitants between 2006 and 2016.

The estimates show that today, the rates of assault and sexual assault are higher in areas that were more male-biased in the past. The coefficient associated with the convict sex ratio is statistically significant at the 5% level for both assault and sexual assault. A one standard deviation increase in the convict sex ratio is associated with an 8.8% increase in the rate of assault and a 12.8% increase in sexual assaults.^30^

We benchmark these effect sizes against the difference in the outcome by high versus low educational attainment (bottom 25% in high school completion rate vs top 25%). The log-difference in assault between high vs low education areas is 0.56, and our coefficient on the convict sex ratio represents 15.7% of this education gap. For sexual offenses, the coefficient on the convict sex ratio represents 23.7 per cent of this gap.

We investigate the long-term consequences of male-biased sex ratios on preventable male mortality in columns 1–3 of Table 3. The dependent variables are the (log) rates of mortality from suicide, broken down by gender, and from prostate cancer. The unit of observation is an LGA. All the results control for the usual historic, geographic, and present-day SA1 controls as well as total male deaths in columns 1 and 3 and total female deaths in column 2. We find strong and robust evidence of elevated rates of male— but not female—suicide and prostate cancer in formerly male-biased areas.

**Table 3:** Historical convict sex ratios and male health

	Preventable mortality				COVID-19 vaccine hesitancy	
	Suicide ln (Incidence per 100K) -Men-	Suicide (top 20 causes of death) -Women-	Prostate cancer ln(Incidence per 100K)	Prostate screening	Will get vaccine -Men-	Will get vaccine -Women-
	(1)	(2)	(3)	(4)	(5)	(6)
Convict sex ratio (z)	0.202***	0.028	0.033***	$-$0.036**	$-$0.039**	$-$0.001
	(0.053)	(0.026)	(0.008)	(0.016)	(0.017)	(0.014)
Spatial HAC *p*-value	0.006	0.235	0.000	0.303	0.058	0.926
Observations	15,600	15,600	15,600	1,349	15,414	15,256
$R^{2}$	0.18	0.25	0.82	0.03	0.06	0.10
Mean of dependent var	69.15	0.17	129.93	0.47	0.78	0.73
Number of clusters	34	34	34	23	31	30
State FE	Yes	Yes	Yes	Yes	Yes	Yes
Geographic controls	Yes	Yes	Yes	Yes	Yes	Yes
Historical controls	Yes	Yes	Yes	Yes	Yes	Yes
Minerals and land type	Yes	Yes	Yes	Yes	Yes	Yes
Present-day SR and pop.	Yes	Yes	Yes	Yes	Yes	Yes

Standard errors clustered at the historical county level. ‘Geographic controls’ are at the postcode level and include the postcodes centroid and the minerals and land type of the postcode. ‘Minerals and land type’ is the presence and type of mineral deposit (major coal; major gold; other) and land formation (plains and plateaus, mountains, other), which are provided by Geoscience Australia. ‘Historic controls’ are: the historical county population, convict population, as well as the proportion of residents working historically in agriculture, domestic service, manufacturing and mining, and government services and learned professions. ‘Present-day SR and population’ are the number of men to women (SR) at the postcode, the total population density of the SA1, whether it is urban, and its population. Demographic data are averages from the 2011 and 2016 Census. In columns 1 and 3, the mean of the dependent variable is reported as the untransformed rate of incidents per 100,000. Column 4 shows whether respondents from the HILDA (males, aged 50+) report to have had a prostate exam in the past 12 months. For columns 5-6, the data come from the Taking the Pulse of the Nation Survey. The question was asked in three waves: (05oct2020–10oct2020) “If a vaccine for COVID-19 is developed and approved for use by the Australian Government, would you be willing to be vaccinated?”; (01feb2021–05jun2021) “Are you willing to have the covid-19 vaccine?”; (14jun2021–23sep2021) This wave asked the previous question with option to answer “I have had the FIRST dose of the vaccine ONLY” or “I have had the first AND second dose of the vaccine”

*$$p<$$ 0.1, **$$p<$$ 0.05, ***$$p<$$ 0.01

The magnitude of the results is large. For suicide—the main cause of death for Australian males under 45 years of age—a one standard deviation increase in the historical convict sex ratio is associated with a 20.2% increase in the male suicide rate. Given a baseline rate of 69.15 (per 100,000), this means that our result corresponds to approximately 14 additional suicide deaths per 100,000. The estimated coefficient corresponds to about 26% of the impact due to the education gap.

For prostate cancer, the most common cancer among men in Australia, the convict sex ratio is associated with a 3.3% increase in deaths, a magnitude corresponding to 8.5% of the education gap. This is likely driven, at least in part, by avoidance of preventative health behavior, in particular prostate cancer screenings which in Australia are recommended for all men over 50. Indeed, the results in column 4 of Table 3 show exactly this. We find that men from historically male-biased areas are 3.6% points (7.7% of the sample mean) less likely to have had a prostate cancer screening in the past 12 months.

Lastly, columns 5 and 6 of Table 3 provide evidence of the relationship between the convict sex ratio and preventative health behavior in the context of the COVID-19 pandemic. Masculinity ideals of strength, invincibility, and help avoidance are often invoked to explain differences between men and women in the takeup of preventative health measures (Springer & Mouzon, 2011) also in the context of COVID-19 vaccination (Capraro & Barcelo, 2020; Steinhauer, 2021). Table 3 shows that a one standard deviation increase in the historical convict sex ratio is associated with a 3.9% point increase in COVID vaccine hesitancy among men (column 5), with no effect on women (column 6). Given the high average vaccination takeup rates in Australia, this represents 18% of the mean vaccine hesitancy among Australian men (which lies at 22% in total).

### Occupational gender segregation

To explore the relationship between historical sex ratios and occupational gender segregation, we regress, separately, the shares of men and women employed in 2011 and 2016 in feminine, neutral, and masculine occupations, as defined in Sect. 4.2. The first (last) three columns of Table 4 present the results for men (women).

**Table 4:** Historical convict sex ratios and occupational gender segregation

	Share of men employed in			Share of women employed in		
	Feminine occupations	Neutral occupations	Masculine occupations	Feminine occupations	Neutral occupations	Masculine occupations
	(1)	(2)	(3)	(4)	(5)	(6)
Convict sex ratio (z)	$-$0.002*	$-$0.005**	0.007***	0.004**	$-$0.005***	0.001
	(0.001)	(0.002)	(0.002)	(0.002)	(0.002)	(0.002)
Spatial HAC *p* value	0.065	0.036	0.017	0.110	0.011	0.425
Observations	16,609	16,609	16,609	16,609	16,609	16,609
$R^{2}$	0.54	0.87	0.86	0.55	0.62	0.36
Mean of dependent var	0.12	0.28	0.59	0.59	0.31	0.10
Number of clusters	34	34	34	34	34	34
State FE	Yes	Yes	Yes	Yes	Yes	Yes
Geographic controls	Yes	Yes	Yes	Yes	Yes	Yes
Historical controls	Yes	Yes	Yes	Yes	Yes	Yes
Minerals and land type	Yes	Yes	Yes	Yes	Yes	Yes
Present-day SR and population	Yes	Yes	Yes	Yes	Yes	Yes

Standard errors clustered at the historical county level. ‘Geographic controls’ are at the postcode level and include the postcodes centroid and the minerals and land type of the postcode. ‘Minerals and land type’ is the presence and type of mineral deposit (major coal; major gold; other) and land formation (plains and plateaus, mountains, other), which are provided by Geoscience Australia. ‘Historic controls’ are: the historical county population, convict population, as well as the proportion of residents working historically in agriculture, domestic service, manufacturing and mining, and government services and learned professions. ‘Present-day SR and population’ are the number of men to women (SR) at the postcode, the total population density of the SA1, whether it is urban, and its population. Demographic data are averages from the 2011 and 2016 Census. Occupations are classified as feminine, neutral, or masculine if their national male share in the occupation is less than 33% (feminine), between 33 and 66% (neutral), or over 66% (masculine)

*$$p<$$ 0.1, **$$p<$$ 0.05, ***$$p<$$ 0.01

Occupational gender segregation could reflect local norms guiding occupational choice as well as local economic conditions. However, the historical sex ratio is not systematically correlated with industrial composition historically (Table 1) nor at an intermediate point in time (1933, see Table 12) nor today (see columns 1 and 2 of Table 11). This suggests that local economic specialization only plays a minimal role. Moreover, to control directly for variation due to local labor market circumstances, we add to our usual covariates a control for total employment in masculine/neutral/feminine occupations at the postcode level. Our main coefficient of interest thus measures residual variation in how much the convict sex ratio explains of the share of workers (by gender) in a specific gender-stereotypical occupation, relative to the share of this occupation in the postcode.

The results paint a striking picture. Historical sex ratios significantly contribute to occupational gender segregation today. The coefficient associated with the convict sex ratio is significant for males across all categories of employment. The sign of the coefficient is consistent with our interpretation that historical sex ratios forged a culture of masculinity, which still leads men to seek employment in stereotypically male occupations, and to shun employment in stereotypically female, and even neutral, occupations.

Overall, a one standard deviation increase in the convict sex ratio is associated with a 0.7% point shift away in the share of men employed in neutral or stereotypically female occupations towards stereotypically male occupations.^31^ We note, however, that the magnitudes here are more modest. For the share of men in masculine professions, our coefficient represents 3.2% of the education gap.

As men shun stereotypically female occupations, women may fill these jobs. Moreover, occupational segregation may not only threaten one’s own gender identity but also imply occupation-specific discrimination against the non-stereotypical sex. In other words, we also expect impacts on female occupational choice. Accordingly, the historical sex ratio is indeed significantly and positively associated with the share of women employed in female occupations (column 4). We now turn to a direct measure of masculinity norms by examining voting in the 2017 same-sex marriage referendum.

### Support for same-sex marriage

Table 5 presents the estimation results using the share of votes in favor of same-sex marriage as the dependent variable in column 1 and the share of abstention in column 2. Abstention can be interpreted as the expression of a weaker form of opposition to same-sex marriage. After all, the status quo at the time of the referendum was that gays and lesbians were not allowed to marry. Abstaining thus meant maintaining that status quo. Indeed, several Members of Parliament who were opposed to same-sex marriage, expressed their intention to abstain and some constituents may have followed suit in this silent opposition.^32^

**Table 5:** Historical convict sex ratios and support for same-sex marriage

	% voted ‘Yes’ (of total registered)	% abstention from referendum	Supports same-sex marriage (HILDA)		
	(1)	(2)	(3)	(4)	(5)
Convict sex ratio (z)	$-$0.022*** (0.006)	0.006** (0.002)	$-$0.056*** (0.017)	$-$0.060*** (0.022)	$-$0.039*** (0.013)
Convict SR $\times$ female				$-$0.009 (0.014)	
Convict SR $\times$ Australia-born					$-$0.021** (0.010)
Spatial HAC *p*-value	0.000	0.010	0.000		
Observations	16,611	16,611	8,838	8,838	8,838
$R^{2}$	0.38	0.33	0.11	0.11	0.11
Mean of dependent var	0.47	0.20	0.61	0.61	0.61
Number of clusters	34	34	29	29	29
State FE	Yes	Yes	Yes	Yes	Yes
Geographic controls	Yes	Yes	Yes	Yes	Yes
Historical controls	Yes	Yes	Yes	Yes	Yes
Minerals and land type	Yes	Yes	Yes	Yes	Yes
Present-day SR and population	Yes	Yes	Yes	Yes	Yes
Individual-level controls	–	–	Yes	Yes	Yes

Same-sex marriage postal survey data are originally at the electorate level and matched to SA1s. The dependent variable in columns (3)-(5) is an indicator variable indicating corresponding to the response to the question: “Homosexual couples should have the same rights as heterosexual couples do”. Positive responses are coded as 1, neutral or negative responses are coded as 0. Source: HILDA waves 2011 and 2015. Individual-level controls include age, gender, and if born in Australia. Standard errors clustered at the historical county level. ‘Geographic controls’ are at the postcode level and include the postcodes centroid and the minerals and land type of the postcode. ‘Minerals and land type’ is the presence and type of mineral deposit (major coal; major gold; other) and land formation (plains and plateaus, mountains, other), which are provided by Geoscience Australia. ‘Historic controls’ are: the historical county population, convict population, as well as the proportion of residents working historically in agriculture, domestic service, manufacturing and mining, and government services and learned professions. ‘Present-day SR and population’ are the number of men to women (SR) at the postcode, the total population density of the SA1, whether it is urban, and its population. Demographic data are averages from the 2011 and 2016 Census

*$$p<$$ 0.1, **$$p<$$ 0.05, ***$$p<$$ 0.01

We express votes and abstention as percentages of total voting population. That is, although “Yes" won more than 60% of all expressed suffrage, it only represented 47% of the total voting population, given the 20% abstention rate. We check the robustness of our results to another measure of attitudes towards same-sex marriage at the individual level from the HILDA survey, in which respondents are asked whether they agree that *"Homosexual couples should have the same rights as heterosexual couples do"* (columns 3 to 5).

The results show that both the share of votes in favor of marriage equality and the participation rate are substantially lower in areas where convict sex ratios were more male-biased in the past. A one standard deviation increase in the convict sex ratio is associated with a 2.2% point decrease in the vote share in favor of same-sex marriage (column 1). This amounts to 4.4% of the mean and corresponds to 39% of the education gap. We also observe that abstention, a lesser form of opposition to same-sex marriage, was significantly higher in areas that were more male biased in the past (column 2).

All of the controls—including all historical controls except for the convict sex ratio, our baseline controls, and the extended set of controls including education and religion—explain 61.1% of the variation in the “Yes” vote. Accounting for the convict sex ratio along with all the other controls explains 70.9% of the “Yes” vote. The convict sex ratio alone thus explains 9.8 percent of the variation in the “Yes” vote, and 25% (= 0.0982/0.3889) of the variation that is unexplained by a wide range of socio-demographic and economic factors, including religious background, unemployment, urbanization, and the present-day sex ratio, as well as historical factors such as total population and economic specialization.

The third column of Table 5 confirms these results with the individual-level survey data. Column 4 shows that men as well as women are more likely to oppose same-sex marriage in areas that were more male biased in the past.^33^ This suggests that both genders have today internalized this norm and may be more likely to transmit it within families, as we investigate in Sect. 7 (where we also discuss the role of migration, cf. column 5).^34^

### Robustness

One might worry that our results (partially) reflect spatial autocorrelation in the residuals (Kelly, 2019). Throughout all tables, we therefore also display HAC-robust standard errors corrected for potential spatial correlation. All the results carry through. In addition, we calculate Moran statistics (a spatial version of the Durbin-Watson statistic) and report the related *p* values in Table 9 for each of our main estimates. These statistics suggest that correlation in spatial noise is limited and unlikely to drive our results. Moreover, we compute *p*-values based on the wild cluster bootstrap-*t* procedure, which accounts for the small number of clusters (Cameron et al., 2008). These *p* values are also reported in Table 9 and indicate that our results are not driven by inappropriate asymptotic assumptions.

We also present treatment effect bounds to gauge the quantitative importance of omitted unobservable factors (Table 9). We follow Oster (2019) and calculate bounds using a maximum R^2^ that is 1.3 times the R^2^ in the specification with all standard observable controls. The bounding set is then defined by the effect in the main specification with standard controls and the treatment effect under the assumption that observables are as important as unobservables. We find that the treatment effects are robust and that the bounding sets exclude zero.

In “Appendix Tables 9 and 10”, we subject our main results to additional robustness tests. Areas that received more male convicts could have followed a different development path in a way that is unrelated to masculinity norms but that could systematically explain our results. For example, if convicts were discriminated against in the labor market, had weaker preferences for education, or held different religious values, these characteristics could in turn have persisted and explain some of our results. We already discussed in Sect. 5 that areas with high versus low convict sex ratios are nowadays statistically indistinguishable from one another in terms of educational achievement, unemployment, and income.

In Table 9, we replicate our baseline results in the odd columns and contrast them with comparable specifications in the even columns that include additional present-day controls at the most granular (SA1) level. These are education (share of the local population that has completed year 12), unemployment rate (by gender), religion shares, median age, median household income, and the proportion of the local population born overseas. To the extent that these variables are endogenous to the convict sex ratio, they are bad controls and might bias our estimates. Table 9 shows that our results are robust to including these additional controls.

Lastly, we assess in “Appendix Table 10” the robustness to controlling for the distance of the SA1 to the nearest port (Panel A) and to controlling for whether an SA1 is part of a metropolitan area (Panel B). In Panel C, we trim the data by removing the two historical counties with the most and the least skewed convict sex ratio. All our results continue to hold.

## Interpretation and mechanisms

So far, we have established a relationship between male-biased sex ratios in the 19th century and present-day outcomes for which we expect masculinity norms to play an important role: violence; suicide and help avoidance; occupational gender segregation; and opposition to sexual minorities’ rights. We now unpack what underlies this long-term relationship. First, we establish that our results reflect the persistent effect of masculinity norms. We do so by ruling out other explanations and by presenting direct evidence that masculinity norms constitute the mechanism that links historical sex ratios to present-day outcomes. Second, we investigate the strength of different persistence mechanisms that may explain the long-term impact of historical sex ratios.

### Interpretation: masculinity norms or other factors?

#### Conservatism

The 2017 referendum on same-sex marriage was a politically charged event. Conservative political parties took position against legalization, and religious organizations were also heavily involved in the campaign. Is the relationship between historical sex ratios and present-day attitudes towards same-sex marriage really specific to attitudes towards homosexuality or merely a reflection of a legacy of sex ratios on social conservatism and political preferences more broadly?

Table 6 shows evidence in favor of the former: broad political attitudes, which go beyond the single issue of rights for homosexuals, are unaffected. Column 1 shows that the coefficient associated with the historical sex ratio does not have a significant effect on the share of votes for conservative parties^35^ in the general election in the year immediately preceding the same-sex marriage referendum. Hence, general conservatism cannot explain our results.

**Table 6:** Alternative mechanisms

	Conservatism	Property crime	China shock	
	Conservative vote share in 2016	log(Incidence per 100K)	Manufacturing share in 1991	Exposure to import shock btw 1992–2016
	(1)	(2)	(3)	(4)
Convict sex ratio (z)	0.006	0.020	0.009	0.147
	(0.012)	(0.030)	(0.008)	(0.126)
Observations	16,611	16,578	14,315	14,315
$R^{2}$	0.21	0.42	0.35	0.35
Mean of dependent var	0.47	3617.64	0.18	0.00
Number of clusters	34	34	31	31
State FE	Yes	Yes	Yes	Yes
Geographic controls	Yes	Yes	Yes	Yes
Historical controls	Yes	Yes	Yes	Yes
Minerals and land type	Yes	Yes	Yes	Yes
Present-day SR and population	Yes	Yes	Yes	Yes

Standard errors clustered at the historical county level. ‘Geographic controls’ are at the postcode level and include the postcodes centroid and the minerals and land type of the postcode. ‘Minerals and land type’ is the presence and type of mineral deposit (major coal; major gold; other) and land formation (plains and plateaus, mountains, other), which are provided by Geoscience Australia. ‘Historic controls’ are: the historical county population, convict population, as well as the proportion of residents working historically in agriculture, domestic service, manufacturing and mining, and government services and learned professions. ‘Present-day SR and population’ are the number of men to women (SR) at the postcode, the total population density of the SA1, whether it is urban, and its population. Demographic data are averages from the 2011 and 2016 Census. The mean of the dependent variable for crime is reported as the un-transformed rate of incidents per 100,000. The China shock variable in column (3) is the percentage of LGA male population employed in manufacturing in 1991 from the Census and in column (4) the LGA-level exposure to import shocks from China by industry (ANZSIC classification, only goods sectors), computed following Autor et al. (2013) using 1991 employment by industry and LGA from the Census and UN Comtrade data by industry from 1992 to 2016. The exposure variable was standardized to have mean 0, sd 1

*$$p<$$ 0.1, **$$p<$$ 0.05, ***$$p<$$ 0.01

#### Crime in general

We argue that the historical sex ratio has forged a locally variegated culture of male violence. Column 2 of Table 6 shows that our earlier results on violent crime and male aggression are not driven by local differences in the prevalence of crime *in general*: the results show that rates of property crime are unrelated to the convict sex ratio.

Cultural underpinnings of violence will act very differently on premeditated versus non-premeditated crime. Assaults are mostly non-premeditated and often result from quickly escalating confrontations, often over what seems to the initiator of the assault as a grave insult to his masculinity or lack of respect (e.g., Wolfgang (1958); Goffman (1959); Wilson and Daly (1985)). Property crime is much more premeditated, less responsive to impulse, and more reflective of a calculation of costs and benefits (Pinker, 2011).

The differentiated long-term effect of sex ratios on assaults versus property crime is, in fact, similar to the situation in the US South, where the Scots-Irish culture of honor still contributes to high rates of homicide and assault, but not to other types of crime, such as property crime (Grosjean, 2014). It is therefore reassuring that we do not find evidence for more widespread crime in areas that were more male-biased in the past, but only evidence on violent crime, one of the costly manifestations of hegemonic masculinity.

#### Industrial composition

One potential mechanism of persistence may be industrial specialization. Although the convict sex ratio was not systematically correlated with industrial composition during the convict era (Table 1), heightened masculinity norms may have influenced industrial composition in the intermediate period. This could then have propagated masculinity norms to the present-day. However, using 1933 census data on employment in 21 industries, we do not find any evidence that convict sex ratios influenced industrial composition in the intermediate period (Table 12).

#### The China shock

In the U.S., the increase in deaths of despair (Case & Deaton, 2020), particularly among men, has been linked to the deterioration in economic circumstances partly caused by rising international manufacturing competition, especially from China (Autor et al., 2013, 2019). One may worry that spatial variation in the sensitivity of local male employment to the rise of China may confound the relationship between historical sex ratios and present-day manifestations of masculinity norms.

To investigate this, we follow Autor et al. (2013) and construct a granular measure of how exposed local male employment was in 1991 to the sudden increase in Chinese imports between 1992 and 2006. The Australian Census allows us to calculate, at the level of Local Government Areas (LGAs), the proportion of men employed in various industries in 1991, at the start of China’s rise to economic prominence.^36^ We then multiply these initial LGA-level shares with subsequent increases in Australia-wide imports from China.

The results in columns 3 and 4 of Table 6 show that male employment in manufacturing in 1991 (column 3) and exposure to import competition from China (column 4) are both unrelated to the convict sex ratio. In line with this orthogonality, Table 11 shows that our main results are robust to controlling for local gender-specific import shocks due to China’s rapid emergence as an economic powerhouse.

#### Institutional differences and legislation

The different states in Australia were independent colonies until 1901. Only New South Wales, Tasmania, and in later periods Western Australia were convict colonies. The colonies became different states today, which vary in their criminal legislation and, until recently, in legislation that affects sexual minorities, in ways that could be correlated with historical circumstances. For example, South Australia, which never harbored convicts, was the first state to decriminalize homosexuality in 1975, and Tasmania the last, in 1997. All our specifications include state fixed effects that remove the influence of time-invariant state characteristics or differences in legislation across states.

#### Convictism

The extent to which present-day violence, crime, and attitudes towards homosexuality are all stained by Australia’s convict past has been the object of a long-standing and intense debate.^37^ Victorian authorities were so concerned about *“blasphemy, rage, mutual hatred, and the unrestrained indulgence of unnatural lust”* among convicts that it became one of the main arguments of transportation abolitionists. This in turn has led some to go as far as stating that: *“prejudice toward LGBTI people [in Australia] can be summed up in one word: convictism”*.^38^

However, we control in all specifications for the number of convicts together with total population, so that our results are immune to any legacy of convictism in and of itself. For assaults and sex offenses, health and suicide, or the share of men employed in male occupations, the coefficient for the number of convicts is not statistically significant. We explore more directly the role played by the share of convicts as a determinant of attitudes towards homosexuality in a short companion paper (Baranov et al., 2020). We show that, contrary to popular opinion, areas with more convicts historically are today more likely to vote in favor of same-sex marriage. This highlights how the convict legacy must be distinguished from that of the radical distortion in sex ratios that convict transportation imposed.

We conclude, having ruled out alternative explanations, that our results reflect how male-biased sex ratios and elevated male-male competition forged a locally variegated culture of male violence, help avoidance, and self-harm, which has persisted until this day. We now turn to additional data that bring more direct evidence that masculinity norms constitute the mechanism that links historical sex ratios to present-day economic, social, and health outcomes.

### Masculinity norms and outcomes: evidence from *ten to men*

This section provides direct evidence on the relationship between masculinity norms and a range of attitudes and behavioral patterns among Australian men. We use data from the Australian Longitudinal Study on Male Health (*Ten to Men*), a study of 16,000 boys and men aged 10 to 55 years at baseline.^39^ The study collects comprehensive data on demographic and socioeconomic characteristics; physical and mental well-being; and health behaviors including the use of health services.

Importantly, the second wave of this survey allows us to construct for each respondent a score on the Conformity to Masculinity Norms Inventory (CMNI-22) and thus gauge the extent to which he adheres to a hegemonic masculine identity.^40^ As discussed in Sect. 2.2, the CMNI is a multi-dimensional scale that measures to what extent an individual man’s actions, thoughts, and feelings conform to hegemonic masculinity norms in Western societies, such as emotional control; risk-taking; violence; dominance; self-reliance; and disdain for homosexuals. To create the CMNI score, *Ten to Men* asks respondents “*Thinking about your own actions, feelings and beliefs, how much do you personally agree or disagree with each statement*”, followed by statements capturing the dimensions in the CMNI-22. Answers range on a four-point Likert scale from 0 (*strongly disagree*) to 3 (*strongly agree*).

“Appendix Table 13” presents correlations between the CMNI-22 score and its primary components of interest. We restrict our sample to adult self-declared heterosexuals (*N*=13,317). The table shows tight correlations, all with the expected sign, between the various expressions of a hegemonic masculinity identity. We find that the strongest correlates of the overall CMNI-22 consist of norms related to dominance (“*I make sure people do as I say*” and “*I love it when men are in charge of women*); disdain for homosexuals (“*It is important to me that people think I am heterosexual*” and “*It would be awful if someone thought I was gay*”); violence (“*Sometimes violent action is necessary*”); and winning (“*Winning is the most important thing*”).

This survey is useful to relate masculinity norms to the outcomes that we study. Table 8 shows how well the overall CMNI-22 score predicts a number of real-life outcomes measured in *Ten to Men*. These correspond closely to the outcomes we have considered (and measured using various other data sources). In column 2, each cell is the coefficient associated with the standardized CMNI-22 score in an OLS regression controlling for respondent age (mean=34.9), Aboriginal or Torres Strait Islander indicator (mean=0.03), marital status (6 categories), language spoken at home (9 categories), as well as state fixed effects. Column 3 shows the coefficient on the CMNI-22 score after also adjusting flexibly for household income, respondent education level, and a socio-economic index based on place of residence.

The results confirm that men who adhere to strict masculinity norms systematically self-report types of behavior that align closely with our behavioral outcomes of interest. In particular, in line with our results in Table 2 on violent assault and sexual offenses, we find that men who score higher on the CMNI-22 scale are significantly more likely to admit they have engaged in intimate partner violence. In line with Table 3, we find that these men are also more likely to have thought about, planned, or attempted to commit suicide and are more likely to display signs of depression (as measured with the standard PHQ-9 Depression Score). They also engage in more risky health behavior, including smoking cigarettes, heavy drinking (*“Injured while drinking”*), and taking hard drugs. In line with medical help avoidance (and our prostate cancer results in Table 3), they are also significantly less likely to have consulted a GP in the past 12 months.

Unfortunately, the *Ten to Men* survey’s geographic coverage is too limited to enable us to relate norms directly to the historical sex ratio. Yet, as explained in Sect. 4.2, we singled out the CMNI dimension that best predicts the behavioral outcomes that we study (see column 4 of Panel A in Table 8) and commissioned the corresponding question on help avoidance to be included in the Australia-wide HILDA survey. In Panel B, we show that areas that were more male-biased in the past, remain characterized today by a greater prevalence of this masculinity norm. To be precise, a one standard deviation increase in the historical sex ratio is associated with a 2.8% decrease at the mean in a man’s inclination to ask for help.

In all, we conclude that male-biased sex ratios instilled strong masculine identities, which then persisted over time and still manifest themselves in a consistent way across political, economic, and social behaviors, attitudes, and norms. We now investigate the persistence mechanisms that underpin these findings.

### Persistence mechanisms

Earlier work on cultural norms discusses two main persistence channels: (i) cultural vertical transmission within families, and (ii) horizontal peer-to-peer socialization (Bisin & Verdier, 2001; Hauk & Saez-Marti, 2002). We investigate each mechanism in turn. First, and consistent with the literature on the transmission of norms about the appropriate conduct and role of women in society (Alesina et al., 2013; Hansen et al., 2015), we find that vertical transmission within families explains part of the persistence of norms about the appropriate conduct of men. Here we also briefly discuss the role of migration. Second, we also document an important role for peer-to-peer transmission in schools.

#### Vertical transmission in families

To investigate vertical transmission, we follow the approach of Nunn and Wantchekon (2011) and GK, and contrast the attitudes of individuals of different ancestries. The idea is that only Australian parents transmit values that reflect historical Australian conditions. Individual-level information on ancestry is only available in the HILDA dataset. We regress individual attitudes towards same-sex marriage on the historical convict sex ratio, a dummy variable that indicates whether the respondent was born in Australia, and an interaction between these two variables. The coefficient associated with the interaction captures the strength of vertical transmission: it measures whether the local historical sex ratio influences more strongly the attitudes of individuals who are born in Australia, compared with foreign-born individuals. We also include the set of standard individual controls.

The results in the last column of Table 5 show that vertical transmission in families plays an important role in explaining the long-term persistent effect of convict sex ratios on attitudes towards same-sex marriage. The coefficient of the interaction term between the local convict sex ratio and whether the respondent was born in Australia is negative and statistically significant at the 5% level. This confirms that attitudes towards homosexuality of individuals born in Australia are indeed more sensitive to the historical sex ratio as compared with individuals born overseas.

#### Migration

The coefficient associated with the main effect of the convict sex ratio in the last column of Table 5 is smaller in magnitude than in our baseline specifications, but still significant at the 1% level. This suggests that, although the local historical sex ratio influences the views of Australian-born more strongly, foreign-born are not insensitive to it.

A recent literature discusses the role of migration in perpetuating cultural equilibria. For example, Bazzi et al. (2020) show that selective migration in and out of frontier areas in the U.S. sustained local norms of individualism. Non-selective migration would, to the contrary, attenuate persistence, as it would dissociate local historical conditions from current ones and bias against finding any relationship between historical conditions and present-day outcomes. However, flows of migrants at any given time are typically marginal as compared with the stock of stayers. This implies that horizontal transmission is more immune to migration, as even non-selected migrants will adjust to local norms.

In the context of international migration, a recent paper by Rapoport et al. (2020) shows, accordingly, that migrants adopt local norms. Our results are compatible with both potential explanations. They can be explained either by selective migration—foreign-born individuals selecting into areas where local opinions are similar to theirs—or by horizontal transmission—migrants adopting local values and attitudes.^41^

#### Horizontal transmission in schools

To investigate horizontal transmission, we focus on peer-to-peer transmission at a young, impressionable age. We use data on bullying in school from LSAC, a longitudinal survey of youths (see Sect. 4). The results in Table 7 show how boys, but not girls, are more likely to be bullied at school in areas that were more male-biased in the past. A one standard deviation increase in the convict sex ratio is associated with a higher likelihood of parents reporting bullying of their sons by 8.5% points. The increase in rates reported by teachers is lower, at 3.6% points, but still statistically significant at the one percent level.

**Table 7:** Horizontal transmission: historical convict sex ratios and bullying in school

	Boys		Girls	
	Bullying reported by teacher	Bullying reported by parents	Bullying reported by teacher	Bullying reported by parents
	(1)	(2)	(3)	(4)
Convict sex ratio (z)	0.036***	0.085***	$-$0.010	0.007
	(0.010)	(0.015)	(0.014)	(0.023)
Spatial HAC *p*-value	0.000	0.000	0.222	0.813
Observations	3,281	3,395	3,178	3,183
$R^{2}$	0.02	0.04	0.01	0.02
Mean of dependent var	0.12	0.30	0.09	0.29
Number of clusters	21	21	22	22
State FE	Yes	Yes	Yes	Yes
Geographic controls	Yes	Yes	Yes	Yes
Historical controls	Yes	Yes	Yes	Yes
Minerals and land type	Yes	Yes	Yes	Yes
Present-day SR and population	Yes	Yes	Yes	Yes
Child-level controls	Yes	Yes	Yes	Yes

Dependent variables are all binary indicators. Standard errors clustered at the historical county level. ‘Geographic controls’ are at the postcode level and include the postcodes centroid and the minerals and land type of the postcode. ‘Minerals and land type’ is the presence and type of mineral deposit (major coal; major gold; other) and land formation (plains and plateaus, mountains, other), which are provided by Geoscience Australia. ‘Historic controls’ are: the historical county population, convict population, as well as the proportion of residents working historically in agriculture, domestic service, manufacturing and mining, and government services and learned professions. ‘Present-day SR and population’ are the number of men to women (SR) at the postcode, the total population density of the SA1, whether it is urban, and its population. Demographic data are averages from the 2011 and 2016 Census. Child individual-level controls include age, gender, and if born in Australia

*$$p<$$ 0.1, **$$p<$$ 0.05, ***$$p<$$ 0.01

Our results on bullying suggest two things. First, they lend credence to the idea that hegemonic masculinity norms are enforced through intimidation, with (perceived) weaker individuals and especially (perceived) homosexuals being likely targets. This can further cement a violent, homophobic and emotionally repressed male social order.^42^ Flood and Hamilton (2008) point out how Australian boys and young men who move outside the boundaries of stereotypically masculine behavior are often verbally and sometimes physically attacked.

**Table 8:** Historical convict sex ratios, masculinity norms and outcomes

	Mean	Coeff. on CMNI (*z*-score)	Coeff. on CMNI with income & education controls	Coeff. on “Bothered ask help” with controls (*z*-score)	Obs
	(1)	(2)	(3)	(4)	(5)
*Panel A: The association between masculinity norms and outcomes, TTM survey*					
Partner violence—frightened partner	0.222	0.038***	0.039***	0.031***	10,286
		(0.004)	(0.004)	(0.004)	
Partner violence—physically hurt partner	0.073	0.024***	0.024***	0.012***	10,286
		(0.003)	(0.003)	(0.003)	
Partner violence—forced partner to have sex	0.016	0.008***	0.009***	0.007***	10,286
		(0.001)	(0.002)	(0.002)	
Suicidal thoughts (lifetime)	0.182	0.018***	0.021***	0.050***	10,296
		(0.004)	(0.004)	(0.004)	
Suicide plan (lifetime)	0.107	0.020***	0.019***	0.033***	10,295
		(0.003)	(0.003)	(0.003)	
Suicide attempt (lifetime)	0.048	0.005**	0.003	0.013***	10,294
		(0.002)	(0.002)	(0.002)	
Currently depressed (PHQ9)	0.060	0.007***	0.010***	0.037***	10,364
		(0.002)	(0.003)	(0.003)	
Injured while drinking	0.156	0.043***	0.041***	0.015***	9,359
		(0.004)	(0.004)	(0.004)	
Smokes cigarettes	0.195	0.022***	0.019***	0.017***	10,291
		(0.004)	(0.004)	(0.004)	
Has used hard drugs	0.289	0.044***	0.038***	0.019***	10,178
		(0.004)	(0.005)	(0.005)	
Consulted GP (past 12 months)	0.826	$-$0.008**	$-$0.008**	$-$0.009**	10,365
		(0.004)	(0.004)	(0.004)	

	Outcome: “Bothered to ask for help”				
	(1)				
*Panel B: Historical convict sex ratios and masculinity norms (“Bothered to ask for help”, HILDA survey)*					
Convict sex ratio (z)	0.084*** (0.027)				
Spatial HAC *p*-value	0.172				
Observations	4,000				
$R^{2}$	0.01				
Mean of dependent var	2.98				
Number of clusters	28				

Panel A summarizes how the CMNI score (columns 2–3) and endorsement of the statement “I am bothered to ask for help” (a component of the CMNI, column 4) predict a set of real-life outcomes. The analysis is based on Ten to Men data, a survey of 16,000 Australian men between 10 and 55 years old. The analysis sample is restricted to self-declared heterosexuals and unweighted. In column 2, each cell is the coefficient associated with the standardized CMNI score in an OLS regression controlling for respondent’s age (mean = 34.908, with 5 missing observations), Aboriginal or Torres Strait Islander indicator (mean = 0.027 with 136 missing observations), marital status (6 categories), and language spoken at home (9 categories). Column 3 and 4 show the coefficient on CMNI score or endorsement of “Bothers to ask for help” after additionally adjusting flexibly for household income, respondent’s education level, and a socio-economic index based on place of residence. Robust standard errors corrected for heteroskedasticity in parentheses. Panel B uses the HILDA 2020 survey to explore how convict sex ratios impact endorsement of one question from the CMNI: “Bothers to ask for help”. Sample is restricted to males and includes the full set of controls as described in Table 5

*$$p<$$ 0.1, **$$p<$$ 0.05, ***$$p<$$ 0.01

Second, they suggest that masculinity norms are perpetuated through horizontal peer pressure, starting at a young age in the playground. This is consistent with List et al. (2019) who find evidence for large peer-level externalities in non-cognitive skills correlated with violence, such as inhibitory control, among boys.^43^ Gilmore (1990) argues in this context that becoming a man is not so much a process of biological maturation, but instead a critical threshold that boys must pass through testing. Much of this testing takes place at school and in the playground.

## Discussion and conclusions

We exploit a historical experiment, convict transportation to Australia in the 18th and 19th century, to identify the long-lasting impact of male-biased sex ratios on masculinity norms and a set of related economic, social, and health outcomes. We find that areas that were heavily male-biased in the past (though not the present) remain characterized by more violent behavior, help avoidance that leads to higher rates of suicide and treatable diseases such as prostate cancer, and a higher likelihood of men selecting more (less) into stereotypically male (female) occupations. We also show that in these areas men were more likely to volunteer for service in World War 1.

Ancillary evidence from the Australian *Ten to Men* survey lends further support for a tight relationship between individuals’ adherence to masculinity norms and the economic, social, and health outcomes we consider in our main analysis. We provide direct evidence that masculinity norms are stronger in areas that were historically more male-biased: support for same-sex marriage is lower, men are more reluctant to ask for help, and are more vaccine hesitant. Taken together, these results indicate that male-biased sex ratios fostered a culture of masculinity that persists until today. Indeed, the consequences of uneven sex ratios have persisted long after contemporary sex ratios returned to their natural rate. We provide suggestive evidence that both socialization within families and male peer pressure at an early age (in the form of bullying in school) contribute to the persistence of such behavioral norms.

While our experimental setting is unique, we believe that our findings have wider applicability. In particular, our results can inform the debate about the long-term socioeconomic consequences and risks of skewed sex ratios as currently observed in many developing countries such as China, India, and parts of the Middle East. In these settings, sex-selective abortion and mortality, polygamy, the cultural relegation and seclusion of women, as well as migration have created societies with skewed effective sex ratios in the marriage market. Our results suggest that the masculinity norms that develop as a result may not only be detrimental to (future generations of) men themselves, but can also have important repercussions for other groups in society, in particular women and sexual minorities.

Our findings also inform discussions about norm setting in heavily male-biased settings *within* societies with otherwise balanced sex ratios, such as the army, police, gender-segregated schools, prisons, management and supervisory boards of large companies, and some academic departments. This is important because we find that the cultural biases due to uneven sex ratios can be both strong and persistent. Our results are thus in line with recent research revealing that decision makers who spent their formative years in all-male high schools or neighborhoods with greater gender inequality, display more gender-biased behavior during their subsequent professional career (Duchin et al., 2021).

In all, our results show how hegemonic masculinity norms and their manifold manifestations can introduce frictions—for example, in the labor market, in health-care systems, and by holding back the socio-economic enfranchisement of sexual minorities—that may contribute to the misallocation of economic resources and, ultimately, dampen economic growth.

### Supplementary Information

Below is the link to the electronic supplementary material.

Supplementary file 1 (pdf 81 KB)

## Figures and Tables

**Fig. 4:**
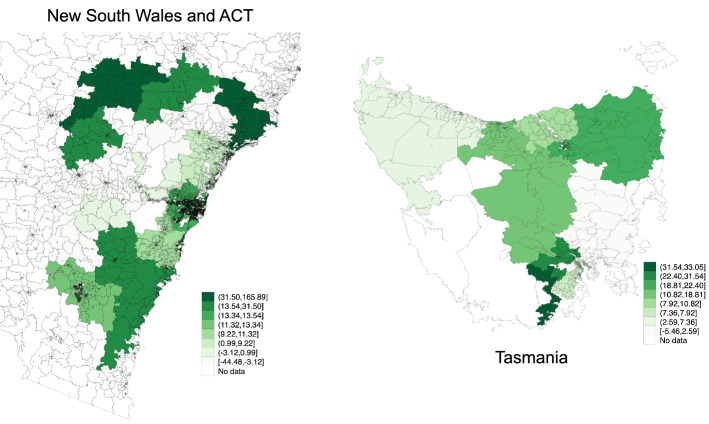
Map of residual convict sex ratios. *Notes:* The maps show the residuals of the Convict Sex Ratio (after controlling for historical industrial composition, population, geographic variables, and present-day population and sex ratio as in our main specification) for the parts of Australia that had convict settlement: Australian Capital Territory, New South Wales, and Tasmania. Boundaries depicted are for the 2016 Statistical Areas Level 1 (SA1), the smallest unit for the release of census data

## Appendix

See Figs. 3, 4, 5 and Tables 9, 10, 11, 12, 13, 14, 15, 16.

**Table 9:** Robustness: controlling for present-day locality covariates

	Assault log (Incidence per 100K)		Sex offenses log (Incidence per 100K)		Suicide log (Incidence per 100K)		Share of men in masculine occupations		% voted ‘Yes’ (of total registered)	
	Standard controls	Extended controls	Standard controls	Extended controls	Standard controls	Extended controls	Standard controls	Extended controls	Standard controls	Extended controls
	(1)	(2)	(3)	(4)	(5)	(6)	(7)	(8)	(9)	(10)
Convict sex ratio (z)	0.088**	0.060**	0.128**	0.104*	0.202***	0.169***	0.007***	0.005***	$-$0.022***	$-$0.013***
	(0.036)	(0.027)	(0.053)	(0.056)	(0.053)	(0.044)	(0.002)	(0.002)	(0.006)	(0.001)
Observations	16,578	16,555	16,578	16,555	15,600	15,580	16,609	16,586	16,611	16,588
$R^{2}$	0.26	0.34	0.59	0.61	0.18	0.25	0.86	0.91	0.38	0.71
Number of clusters	34	34	34	34	34	34	34	34	34	34
Moran statistic *p*-value	0.369	–	0.104	–	0.369	–	0.188	–	0.116	–
Wild cluster bootstrap *p*-value	0.038	–	0.082	–	0.002	–	0.012	–	0.022	–
Bounds on the treatment effect (Delta=1, Rmax=1.3*R)	(0.088, 0.478)	–	(0.128, 0.905)	–	(0.184, 0.202)	–	(0.006, 0.007)	–	($-$0.091, $-$0.022)	–
State FE	Yes	Yes	Yes	Yes	Yes	Yes	Yes	Yes	Yes	Yes
Geographic controls	Yes	Yes	Yes	Yes	Yes	Yes	Yes	Yes	Yes	Yes
Historical controls	Yes	Yes	Yes	Yes	Yes	Yes	Yes	Yes	Yes	Yes
Minerals and land type	Yes	Yes	Yes	Yes	Yes	Yes	Yes	Yes	Yes	Yes
Present-day SR and population	Yes	Yes	Yes	Yes	Yes	Yes	Yes	Yes	Yes	Yes

Standard errors clustered at the historical county level. ‘Geographic controls’ are at the postcode level and include the postcodes centroid and the minerals and land type of the postcode. ‘Minerals and land type’ is the presence and type of mineral deposit (major coal; major gold; other) and land formation (plains and plateaus, mountains, other), which are provided by Geoscience Australia. ‘Historic controls’ are: the historical county population, convict population, as well as the proportion of residents working historically in agriculture, domestic service, manufacturing and mining, and government services and learned professions. ‘Present-day SR and population’ are the number of men to women (SR) at the postcode, the total population density of the SA1, whether it is urban, and its population. Demographic data are averages from the 2011 and 2016 Census. ’Present-day SA1 controls’ include education (share completed year 12), unemployment rate (by gender), religion shares, median age, median household income, and proportion born overseas at the SA1 level. Wild cluster bootstrap *p*-values are computed using 1,000 replications following Cameron et al. (2008). Bounds on the treatment effect are computed using the method developed by Oster (2019) and using a maximum R2 of 1.3 times the R2 in the specification with all our standard observable controls

*$$p<$$ 0.1, **$$p<$$ 0.05, ***$$p<$$ 0.01

**Table 10:** Additional robustness tests

	Assault	Sex offenses	Suicide	Share of men in masculine occupations	% voted ‘Yes’
	(1)	(2)	(3)	(4)	(5)
*Panel A: Controlling for distance to port*					
Convict sex ratio (z)	0.046*	0.107**	0.239***	0.007**	$-$0.015***
	(0.027)	(0.049)	(0.084)	(0.003)	(0.004)
Observations	16,578	16,578	15,600	16,609	16,611
$R^{2}$	0.30	0.61	0.23	0.88	0.40
Number of clusters	34	34	34	34	34
*Panel B: Controlling for metropolitan areas*					
Convict sex ratio (z)	0.087**	0.134**	0.201***	0.007***	$-$0.022***
	(0.036)	(0.064)	(0.052)	(0.002)	(0.006)
Observations	16,578	16,578	15,600	16,609	16,611
$R^{2}$	0.26	0.59	0.18	0.86	0.38
Number of clusters	34	34	34	34	34
*Panel C: Dropping outliers in SR (trimming 1 from top and bottom)*					
Convict sex ratio (z)	0.141**	0.153**	0.253***	0.006**	$-$0.030***
	(0.052)	(0.073)	(0.062)	(0.003)	(0.008)
Observations	16,142	16,142	15,164	16,173	16,175
$R^{2}$	0.28	0.59	0.18	0.86	0.37
Number of clusters	32	32	32	32	32

Standard errors clustered at the historical county level. ‘Geographic controls’ are at the postcode level and include the postcodes centroid and the minerals and land type of the postcode. ‘Minerals and land type’ is the presence and type of mineral deposit (major coal; major gold; other) and land formation (plains and plateaus, mountains, other), which are provided by Geoscience Australia. ‘Historic controls’ are: the historical county population, convict population, as well as the proportion of residents working historically in agriculture, domestic service, manufacturing and mining, and government services and learned professions. ‘Present-day SR and population’ are the number of men to women (SR) at the postcode, the total population density of the SA1, whether it is urban, and its population. Demographic data are averages from the 2011 and 2016 Census

*$$p<$$ 0.1, **$$p<$$ 0.05, ***$$p<$$ 0.01

**Table 11:** Robustness to the China shock

	Assaults controlling for China exposure	Male suicide controlling for China exposure	Masculine occupation share controlling for China exposure
	(1)	(2)	(3)
Convict sex ratio (z)	0.081**	0.163**	0.005*
	(0.034)	(0.072)	(0.003)
Observations	14,303	14,315	14,314
$R^{2}$	0.29	0.19	0.87
Mean of dependent var	7.23	3.94	0.60
Number of clusters	31	31	31
State FE	Yes	Yes	Yes
Geographic controls	Yes	Yes	Yes
Historical controls	Yes	Yes	Yes
Minerals and land type	Yes	Yes	Yes
Present-day SR and pop	Yes	Yes	Yes

The table show results on assault, male suicide, and share of men working in masculine professions while controlling for LGA-level exposure to import shocks from China by industry (ANZSIC classification, only goods sectors), computed following Autor et al. (2013) using 1991 employment by industry and LGA from the Census and UN Comtrade data by industry from 1992 to 2016. Standard errors clustered at the historical county level. ‘Geographic controls’ are at the postcode level and include the postcodes centroid and the minerals and land type of the postcode. ‘Minerals and land type’ is the presence and type of mineral deposit (major coal; major gold; other) and land formation (plains and plateaus, mountains, other), which are provided by Geoscience Australia. ‘Historic controls’ are: the historical county population, convict population, as well as the proportion of residents working historically in agriculture, domestic service, manufacturing and mining, and government services and learned professions. ‘Present-day SR and population’ are the number of men to women (SR) at the postcode, the total population density of the SA1, whether it is urban, and its population. Demographic data are averages from the 2011 and 2016 Census

*$$p<$$ 0.1, **$$p<$$ 0.05, ***$$p<$$ 0.01

**Table 12:** 1933 population, industrial composition, and convict sex ratios

	Coefficient on Convict Sex Ratio				
	$\beta$	standard error	*p* value	Mean of dependent variable	N
	(1)	(2)	(3)	(4)	(5)
Total population (ln)	$-$0.01	(0.05)	0.80	1.88	162
Total males employed (ln)	$-$0.01	(0.05)	0.84	7.77	162
Fishing and trapping (ln)	$-$0.12	(0.09)	0.19	2.35	162
Wheat farming (ln)	0.38	(0.20)	0.08*	1.17	162
Fruit growing (ln)	$-$0.21	(0.17)	0.24	2.01	162
Farming (mixed) (ln)	$-$0.10	(0.10)	0.32	4.37	162
Agriculture grazing (ln)	$-$0.18	(0.12)	0.16	3.38	162
Agriculture dairy (ln)	$-$0.22	(0.14)	0.13	2.87	162
Agriculture (other) (ln)	$-$0.04	(0.08)	0.63	3.74	162
Forestry (ln)	0.28	(0.18)	0.14	2.45	162
Mining (ln)	$-$0.01	(0.20)	0.95	3.28	162
Manufacturing (ln)	0.09	(0.09)	0.35	5.38	162
Building (ln)	0.08	(0.06)	0.21	4.31	162
Roads and rail (ln)	$-$0.01	(0.07)	0.93	5.39	162
Gas, water, electric (ln)	$-$0.08	(0.10)	0.42	2.81	162
Land transportation (ln)	0.03	(0.05)	0.58	4.71	162
Water transportation (ln)	0.12	(0.10)	0.26	2.44	162
Communication (ln)	0.07	(0.04)	0.06*	3.35	162
Commerce and finance (ln)	0.07	(0.06)	0.29	5.41	162
Public administration (ln)	0.01	(0.05)	0.82	4.99	162
Entertainment and sport (ln)	0.04	(0.10)	0.68	2.45	162
Domestic service (ln)	0.03	(0.03)	0.23	5.21	162

Data are from 1933 Census at the LGA level. Outcome variables are listed in the first column. Each row corresponds to a regression of the outcome variable on convict ratio, following the specification in the rest of the paper (including Geographic, Minerals and land types, and historic controls). Regressions also control for contemporaneous (1933) population and sex ratio, except for the first row where the outcome is population. Standard errors clustered at the historical county level

*$$p<$$ 0.1, **$$p<$$ 0.05, ***$$p<$$ 0.01

**Table 13:** The conformity to masculinity norms inventory (CMNI) and its main components

	CMNI	(01)	(02)	(03)	(04)	(05)	(06)	(07)	(08)	(09)	(10)	(11)	(12)	(13)	(14)
CMNI	1.00														
(01) - People do as I say	0.41$*$	1.00													
(02) - Awful if thought gay	0.37$*$	0.15$*$	1.00												
(03) - Men in charge of women	0.47$*$	0.24$*$	0.27$*$	1.00											
(04) - Talk about feelings	$-$0.32$*$	0.01	$-$0.07$*$	$-$0.04$*$	1.00										
(05) - Important thought of as heterosexual	0.39$*$	0.14$*$	0.58$*$	0.24$*$	$-$0.02$*$	1.00									
(06) - Violence never justified	$-$0.37$*$	$-$0.01	0.05$*$	$-$0.07$*$	0.11$*$	0.04$*$	1.00								
(07) - Share feelings	$-$0.32$*$	0.01	$-$0.05$*$	$-$0.04$*$	0.75$*$	$-$0.01	0.13$*$	1.00							
(08) - Hate to be important	$-$0.18$*$	$-$0.05$*$	0.07$*$	0.02$*$	$-$0.06$*$	0.03$*$	0.06$*$	$-$0.05$*$	1.00						
(09) - Violent action necessary	0.41$*$	0.06$*$	0.02$*$	0.14$*$	$-$0.07$*$	0.05$*$	$-$0.47$*$	$-$0.08$*$	$-$0.01	1.00					
(10) - Not bothered by losing	$-$0.36$*$	$-$0.12$*$	$-$0.06$*$	$-$0.09$*$	0.06$*$	$-$0.07$*$	0.09$*$	0.06$*$	0.16$*$	$-$0.05$*$	1.00				
(11) - Never ask for help	0.25$*$	0.02$*$	0.05$*$	0.05$*$	$-$0.23$*$	0.04$*$	$-$0.00	$-$0.23$*$	0.15$*$	0.03$*$	0.01	1.00			
(12) - Enjoy risks	0.35$*$	0.10$*$	$-$0.03$*$	0.07$*$	0.04$*$	0.02$*$	$-$0.12$*$	0.05$*$	$-$0.11$*$	0.15$*$	$-$0.05$*$	0.00	1.00		
(13) - Winning most important	0.49$*$	0.25$*$	0.15$*$	0.24$*$	$-$0.03$*$	0.15$*$	$-$0.06$*$	$-$0.02	$-$0.10$*$	0.09$*$	$-$0.36$*$	0.06$*$	0.15$*$	1.00	
(14) - Bothered by asking for help	0.34$*$	0.05$*$	0.09$*$	0.08$*$	$-$0.20$*$	0.09$*$	$-$0.06$*$	$-$0.20$*$	0.10$*$	0.10$*$	$-$0.08$*$	0.49$*$	0.02$*$	0.14$*$	1.00

This table presents raw correlations between the CMNI score and its primary components of interest. The analysis is based on a nation-wide survey (Ten to Men), with oversampling in rural and remote areas, of 16,000 Australian men between 10 and 55 years old (Bandara et al., 2019). For each component, respondents are asked: *“Thinking about you own actions, feelings and beliefs, how much do you personally agree or disagree”* with each statement, followed by statements capturing the several dimensions in the CMNI. Possible answers are on a scale from 0 to 3 (0= Strongly disagree; 1 = Disagree; 2 = Agree; 3 = Strongly agree). The analysis sample is restricted to self-declared heterosexuals (N=13,317) and unweighted

*$$p < 0.05$$

**Table 14:** Robustness: excluding present-day controls

	Assault log(Incidence per 100K)	Sex offenses log(Incidence per 100K)	Suicide log(Incidence per 100K)	Share of men in masculine occupations	% voted ‘Yes’ (of total registered)
	(1)	(2)	(3)	(4)	(5)
Convict sex ratio (z)	0.091**	0.107*	0.200***	0.008***	$-$0.022***
	(0.041)	(0.055)	(0.051)	(0.002)	(0.007)
Observations	16,578	16,578	15,600	16,609	16,611
$R^{2}$	0.20	0.56	0.18	0.83	0.37
Number of clusters	34	34	34	34	34
State FE	Yes	Yes	Yes	Yes	Yes
Geographic controls	Yes	Yes	Yes	Yes	Yes
Historical controls	Yes	Yes	Yes	Yes	Yes
Minerals and land type	Yes	Yes	Yes	Yes	Yes
Present-day SR and population	No	No	No	No	No

Standard errors clustered at the historical county level. ‘Geographic controls’ are at the postcode level and include the postcodes centroid and the minerals and land type of the postcode. ‘Minerals and land type’ is the presence and type of mineral deposit (major coal; major gold; other) and land formation (plains and plateaus, mountains, other), which are provided by Geoscience Australia. ‘Historic controls’ are: the historical county population, convict population, as well as the proportion of residents working historically in agriculture, domestic service, manufacturing and mining, and government services and learned professions. ‘Present-day SR and population’ are the number of men to women (SR) at the postcode, the total population density of the SA1, whether it is urban, and its population. Demographic data are averages from the 2011 and 2016 Census

*$$p<$$ 0.1, **$$p<$$ 0.05, ***$$p<$$ 0.01

**Table 15:** IV specification

	Assault log(Incidence per 100K)	Sex offenses log(Incidence per 100K)	Suicide log(Incidence per 100K)	Share of men in masculine occupations	% voted ‘Yes’ (of total registered)
	(1)	(2)	(3)	(4)	(5)
Historical sex ratio	0.112**	0.163***	0.263***	0.009***	$-$0.028**
	(0.054)	(0.057)	(0.072)	(0.003)	(0.013)
Observations	16,578	16,578	15,600	16,609	16,611
$R^{2}$	0.26	0.59	0.18	0.86	0.36
Mean of dependent var					
Number of clusters	34	34	34	34	34
F-statistic (1st stage)	15	15	16	17	15
State FE	Yes	Yes	Yes	Yes	Yes
Geographic controls	Yes	Yes	Yes	Yes	Yes
Historical controls	Yes	Yes	Yes	Yes	Yes
Minerals and land type	Yes	Yes	Yes	Yes	Yes
Present-day SR and population	Yes	Yes	Yes	Yes	Yes

Historical sex ratio is instrumented using convict sex ratio. Standard errors clustered at the historical county level. ‘Geographic controls’ are at the postcode level and include the postcodes centroid and the minerals and land type of the postcode. ‘Minerals and land type’ is the presence and type of mineral deposit (major coal; major gold; other) and land formation (plains and plateaus, mountains, other), which are provided by Geoscience Australia. ‘Historic controls’ are: the historical county population, convict population, as well as the proportion of residents working historically in agriculture, domestic service, manufacturing and mining, and government services and learned professions. ‘Present-day SR and population’ are the number of men to women (SR) at the postcode, the total population density of the SA1, whether it is urban, and its population. Demographic data are averages from the 2011 and 2016 Census

*$$p<$$ 0.1, **$$p<$$ 0.05, ***$$p<$$ 0.01

**Table 16:** OLS specification using historical population-wide sex ratio

	Assault log(Incidence per 100K)	Sex offenses log(Incidence per 100K)	Suicide log(Incidence per 100K)	Share of men in masculine occupations	% voted ‘Yes’ (of total registered)
	(1)	(2)	(3)	(4)	(5)
Historical SR (*z*)	0.035	0.151**	0.274**	0.004	$-$0.004
	(0.059)	(0.061)	(0.100)	(0.004)	(0.011)
Observations	16,578	16,578	15,600	16,609	16,611
$R^{2}$	0.26	0.59	0.18	0.86	0.37
Number of clusters	34	34	34	34	34
State FE	Yes	Yes	Yes	Yes	Yes
Geographic controls	Yes	Yes	Yes	Yes	Yes
Historical controls	Yes	Yes	Yes	Yes	Yes
Minerals and land type	Yes	Yes	Yes	Yes	Yes
Present-day SR and population	Yes	Yes	Yes	Yes	Yes

Independent variable is historical sex ratio. Standard errors clustered at the historical county level. ‘Geographic controls’ are at the postcode level and include the postcodes centroid and the minerals and land type of the postcode. ‘Minerals and land type’ is the presence and type of mineral deposit (major coal; major gold; other) and land formation (plains and plateaus, mountains, other), which are provided by Geoscience Australia. ‘Historic controls’ are: the historical county population, convict population, as well as the proportion of residents working historically in agriculture, domestic service, manufacturing and mining, and government services and learned professions. ‘Present-day SR and population’ are the number of men to women (SR) at the postcode, the total population density of the SA1, whether it is urban, and its population. Demographic data are averages from the 2011 and 2016 Census

*$$p<$$ 0.1, **$$p<$$ 0.05, ***$$p<$$ 0.01

## Footnotes

1 The concept of hegemonic masculinity was in fact first defined in Australia, before it became a central concept of gender studies in the U.S. and elsewhere. R.W. Connell, an Australian sociologist, first proposed the concept of hegemonic masculinity in reports from a field study of Australian high schools in a conceptual discussion of the making of masculinities (Connell et al., 1982).

2 The index was developed by Mahalik et al. (2003) together with a larger group of researchers in counseling psychology. It was validated through focus groups, pilot studies, and clinical studies. The 11 factors subscale was extracted from 144 original items following a factor analysis. Reported alphas in the original study were 0.94 for the total CMNI and between 0.72 and 0.91 for the 11-item subscale. The total CMNI score and the 11-items subscale were shown to correlate with other normative measure of masculinity, measures that assessed conflict and stress associated with masculine norms, and measures of attitudes toward psychological help-seeking, psychological distress, and social desirability. Mahalik et al. (2003) explain each of the 11 items in more detail: “*Conformity to Winning* should relate to wanting to be admired and respected, successful/powerful/competitive, performing competently, and being physically adequate. *Conformity to Emotional Control* should relate to other measures of emotional restriction. *Risk-Taking* should relate to measures of toughness and adventure. *Violence* should relate to measures of toughness and violence. *Power Over Women* should relate to anti-femininity and subordinating women. *Dominance* should relate to wanting to be admired and respected, tough, successful/powerful/competitive, and subordinating women. *Playboy* should relate to adventure, anti-femininity, concealing emotions, and subordinating women. *Self-Reliance* should relate to disconnection from others, and in terms of disconnection as measured by the other masculinity scales, this should relate to emotional disconnection. *Primacy of Work* should relate to being a breadwinner, enduring work like a machine, pursuing success, and experiencing conflict between work and family/school obligations. *Disdain for Homosexuals* should relate to anti-femininity and restricting one’s affectionate behavior with other men. *Pursuit of Status* should relate to being a breadwinner, admired and respected, successful/powerful/competitive, and performing well” (Mahalik et al., 2003, p.14).

3 In our data, rates of assault are indeed strongly correlated with rates of domestic and sexual violence. The correlation coefficients between, on the one hand, the (log) rate of (non-domestic) assaults (in which the majority of victims are male) and the (log) rates of domestic violent assaults and sexual violence (in which the majority of victims are female) are 0.89 and 0.88, respectively.

4 Attanasio and Lechene (2002). See also Fernández et al. (2021) specifically for attitudes of women towards same-sex marriage.

5 Fear of a ‘convict stain’ emerged during the anti-transportationist movement in the mid-1850s (Holdridge, 2015).

6 Our findings that circumstances prevailing at the time of convict settlement of Australia have persistent effects in the long-run align with a literature that highlights how cultural norms originate in critical junctures in history (Nunn & Wantchekon, 2011; Grosfeld et al., 2013; Galor & Özak, 2016) and on how founder populations leave persistent identities (Grosjean, 2014; Bazzi et al., 2020; Haddad, 2022).

7 Our emphasis on within-sex competition also follows an extensive literature in biology (Bachtrog et al., 2014) and evolutionary psychology (Buss, 2016) on the sex ratio (the number of males relative to females) as the primary driver of male-male competition and of behavioral differences between the sexes, including male aggressiveness, excessive risk taking, and dominant behavior over lower-ranked males and females. Intrasexual competition also applies to women and may explain another range of behaviors, as suggested by Blake et al. (2018).

8 Wei and Zhang (2011) consider male-male competition over wives as a motive for savings in the context of male-biased sex ratios. Recently, Alger (2021) considers a theoretical evolutionary model of male competitiveness and polygyny rates, although the model does not allow for skewed sex ratios.

9 Although most papers find a positive association between male-biased sex ratios and crime and violence, some document a negative relationship (Schacht et al., 2016) A possible reason for these ambiguous results is that the variation in sex ratios exploited in these papers results from sex-selective migration, abortion, or mortality (Hesketh & Xing, 2006)—which are themselves endogenous cultural outcomes (Qian, 2008; Almond & Mazumder, 2011; Carranza, 2014; Xue, 2016)—or from incarceration (Schacht et al., 2016), another endogenous confound. In contrast, we rely on a natural experiment that generated quasi-random variation in the sex ratio. Our results confirm the existence of a positive relationship between sex ratios and crime.

10 At an aggregate level, countries with English common law, a communist past, or high (contemporary) sex ratios are less accepting of homosexuality (Asal et al., 2013; Andersen & Fetner, 2008; Chang, 2021) These studies do not address the potential endogeneity of such cross-country differences. Aksoy et al. (2020) exploit the gradual rollout of same-sex relationship recognition throughout Europe to demonstrate how laws can shape attitudes towards sexual minorities.

11 See i.a. Ullman and Fidell (1989) and Hesketh and Xing (2006) and the related literature cited in the Introduction.

12 Parental investment theory advances that from an evolutionary perspective the potential reproductive benefits from promiscuity and multiple mating are higher for men than for women (Symons, 1979; Buss, 2016) Although human males are often involved in provisioning and parenting, their effort is on average both lower and more variable than that of their female partners in most, if not all, cultures (Hrdy, 2011). Paternal provisioning and parenting are aligned with females’ interest since they raise the welfare of their offspring (Hrdy, 2011).

13 In Sect. 7.1.3, we present detailed CMNI-based survey data from Australia and show that the extent to which individual men adhere to hegemonic masculinity norms is indeed highly predictive of real-world outcomes related to violence, risk taking, unhealthy behavior, suicidal tendencies, and help avoidance.

14 Many men who conform to hegemonic masculinity norms are put off by the prospect of an invasive screening procedure, also because of the perceived homosexual associations of a digital rectal examination. Moreover, these men often fear that a diagnosis of prostate cancer and a possible prostatectomy may cause sexual dysfunction and impotence and hence threaten their manhood. See James et al. (2017) and the references therein.

15 A second but related mechanism that may underlie the relationship between sex ratios and attitudes towards homosexuality is that men tend to be more hostile to homosexuality than women (Kite, 1984; Britton, 1990; Winegard et al., 2016) In regions with high sex ratios (that is, an abundance of men) hostility against homosexuals is thus more likely to become the dominant norm. This effect can be particularly strong in settings, such as the Victorian era, in which men hold significantly more power than women in determining social norms and laws (Guttentag & Secord, 1983). Relatedly, to the extent that skewed sex ratios led to an increase in situational homosexual activity, and such same-sex relations were actively repressed and punished by the colonial authorities, this may have further cemented the relationship between convict sex ratios and negative present-day attitudes towards homosexuality.

16 In total, five convicts were ever transported to Australia for ‘culpable homicide’ and 141 for ‘murder’. 113 were deported for ‘stealing a handkerchief’, 189 for ‘stealing a watch’, 191 for ‘pickpocketing’, and 732 for ‘steeling a sheep’. These statistics are obtained from convict records and are available at convictrecords.com.au/crimes (accessed 16 March 2018). These data were digitized from the British convict transportation registers, which contain information on the characteristics of each convict in each shipment but not on where such convicts were assigned once in Australia.

17 The rest of the (white) population consisted of colonial administrators, ex-convicts, free migrants as well as people born in the colony, of all ages.

18 Another government official stated that *“the horrible crime which brought down fire from heaven on those devoted cities mentioned in scripture, exists and is practiced here to a great extent”* (Norton, 2016) while Reverend John West asked in the Launceston Examiner (1846) *“Shall Tasman’s Isle so famed, so lovely and so fair, from other nations be estranged, the name of Sodom bear?”* Interestingly, these moral concerns of the Australian as well as the English elite went all the way up to British Parliament and eventually became a driving force behind the anti-transportationist movement in the 1840s and 1850s.

19 It was only during the last decades of the 19th century, that colonial society (and in particular its judiciary) started to acknowledge homosexual identity as a sexual orientation, rather than just viewing sodomy as an aberrant act (Fogarty, 1992; Aldrich, 2008).

20 This is the second oldest Census for New South Wales. The 1833 Census lacks sufficient geographic granularity for our purpose.

21 The dates of the Censuses vary because states were independent colonies until 1901.

22 “Counties” is used here to refer to historical administrative divisions within the different colonies of Australia, variously called “counties”, “police districts”, “towns”, or “districts”.

23 The First Infantry Brigade was supplied by New South Wales and the Second by Victoria. Queensland supplied the 9th Infantry Battalion of the 3d Infantry Brigade, South Australia the 10th Infantry Battalion of the 3d Infantry Brigade, Western Australia the 11th Infantry Battalion of the 3d Infantry Brigade; and Tasmania half of the 12th Infantry Battalion of the 3rd Infantry Brigade (with the other half supplied by South and Western Australia).

24 The 1933 Census also recorded female veterans, but there were very few such cases. We find no effect of convict sex ratio on female veterans, but given their rarity, this result is not surprising.

25 The question read ‘*If a vaccine for COVID-19 is developed and approved for use by the Australian government, would you be willing to be vaccinated?*’ or, in later waves, ‘*Are you willing to have the COVID-19 vaccine?*’.

26 The only outcome that is marginally correlated with gender norms is attitudes towards same-sex marriage.

27 Results are similar when excluding any present-day controls (Table 14).

28 The population sex ratio in this context includes convicts as well as emancipists (ex-convicts), colonial administrators, free migrants, and white people born in the colony; Aborigines being excluded from the colonial Censuses. The population sex ratio differed from the convict sex ratio due to fertility, mobility and self-selection, and so it is a more endogenous measure. Nevertheless, it may be informative to compare the results using the convict sex ratio versus the population sex ratio, which we show in Table 16. Using the overall sex ratio provides qualitatively similar but not identical results. While effect sizes for sexual offenses and suicide are nearly identical to those when using the convict ratio, the effects on assaults, occupational gender segregation and voting in the same-sex marriage referendum are muted by comparison to using the convict ratio. This implies that selection effects offset effects of skewed sex ratios particularly for these outcomes.

29 We do not show those results as they are nearly identical. This is not surprising given that the correlation coefficient between total convict number and total convict men is 0.99.

30 According to a more detailed breakdown of assaults by gender that we were able to obtain for New South Wales, 83% of assaults are committed by men and 72% of the victims are male. This variable thus broadly proxies for male-on-male violence.

31 The sum of the two point estimates for female and neutral occupations—0.002 and 0.005, respectively—corresponds to the estimate for the share of men in masculine occupations (0.007).

32 Most members of the Liberals/Nationals coalition who were the most prominent opponents to same-sex marriage abstained during the vote for the final bill that legalized same-sex marriage.

33 In unreported regressions, we extend this analysis by including additional waves, going back to 2005. The coefficients based on each wave are similar to those reported in Table 5. Additionally, given that the HILDA is a panel dataset, we constructed an individual change in attitudes towards LGBT and use that as our outcome. We find that the historical convict sex ratio appears unrelated to the recent evolution of norms, as the related coefficient is small and statistically insignificant. This suggests that the gradual increase in support for LGBT people was relatively uniform across Australia and did not involve much convergence between, on one hand, areas with (historically determined) low initial support for same-sex minorities and, on the other hand, the rest of the country.

34 Masculinity norms, like gender norms about women, are social norms that can transmit vertically (within the family) and horizontally (among peers). They can therefore be held by both men and women and may affect the behavior and social preferences of both (Reny, 2020).

35 Australia is by and large characterized by a two-party system, consisting of a socially conservative and economically liberal Liberal-National Coalition and a more socially progressive Labour Party. The dependent variable in column 1 is the share of votes for the Liberal-National Coalition in the 2016 general election.

36 In principle, we can disaggregate local male employment at the level of 21 ANZSIC (Australian and New Zealand Standard Industrial Classification) economic sectors. It turns out that 17 of these are services sectors and, according to the UN Comtrade database, Australia did not import these services from China. We therefore focus on four main ANZSIC sectors—manufacturing; mining; agriculture-fishing-forestry; and ICT. Importantly, the emergence of China not only led to a sharp increase in manufacturing imports from China to Australia (equivalent to what happened in the US). China’s emergence also involved fast growth in the exports of mined minerals to China. This means that the worst hit areas in terms of male employment reduction, were those with a lot of pre-shock manufacturing but little mining. Some other areas, those with little ex ante manufacturing employment but many men employed in mining, may in fact have benefited from China’s emergence.

37 See https://theconversation.com/stain-or-badge-of-honour-convict-heritage-inspires-mixed-feelings-41097.

38 See https://www.theguardian.com/commentisfree/2017/sep/30/australias-homophobia-is-deeply-rooted-in-its-colonial-past.

39 The survey is oversampled in rural and remote areas. Sampling and other survey methods are described in more detail in Bandara et al. (2019). While the *Ten to Men* survey contains geographic identifiers, so that respondents can be linked to SA1 areas, the survey only overlaps with 11 out of the 34 historical counties with convicts. For this reason, we cannot analyze directly the impact of historical sex ratios on the CMNI-22 using the empirical framework we have used so far.

40 The CMNI-22 is a shorter version of the original 94-item CMNI as developed by Mahalik et al. (2003) and uses the two highest loading items for each of the 11 factors from the original study.

41 Unfortunately, our data do not allow us to disentangle these mechanisms further by measuring how individual migration decisions correlate with individual attitudes and characteristics.

42 LGBTQ youths are at much higher risk of bullying in schools, with two thirds of LGBTQ young people reporting school bullying (Guasp, 2012).

43 While it is plausible that bullying among boys at school perpetuates masculinity norms, such behavior may to some extent also be a mere expression of (vertically transmitted) norms.
