# Supplementary material for: Men. Male-biased sex ratios and masculinity norms: evidence from Australia’s colonial past
Source: J Econ Growth (Boston). 2023 Apr 1:1–58. Online ahead of print. doi: 10.1007/s10887-023-09223-x (PMC10067017; doi:10.1007/s10887-023-09223-x)
Supplement: Supplementary file 1 — (pdf 81 KB) [file 10887_2023_9223_MOESM1_ESM.pdf]

Online Appendix

Male-biased Sex Ratios and Masculinity Norms: Evidence from Australia's Colonial Past, by Baranov, De Haas, Grosjean

A Variable description

Below we describe the data sources and definitions of the variables used in the paper. The table below summarizes the data sources used, the unit of reporting, and the unit of reporting, and the number of observations in the original unit of reporting being used in the analysis.

Data source	Unit of reporting	Observations in original unit
A.1 Historical data	Historical county	34
A.2 1933 census	Historical LGA	162
A.3 Minerals/land formation/soil	Postcode	515
A.4 Census (2011 and 2016)	SA1 level	16,611
A.5 Crime	Postcode	515
A.6 Mortality	Local Government Area (LGA)	106
A.4 Occupations (Census 2011 and 2016)	Postcode	515
A.7 Same-sex marriage referendum	Electoral Division	50
A.8 HILDA survey (2011, 2016 waves)	Individual (merged at SA1)	8,826
A.9 LSAC survey (waves 2004-2014)	Individual (merged at Postcode)	6,763
A.10 Election voting	Postcode	515
A.11 Vaccine hesitancy	Postcode	368

A.1 Historical variables

Our data to calculate historical sex ratios is based on the earliest reliable Census in each state, which we take from the Historical Census and Colonial Data Archive (HCCDA). In all colonies, except for New South Wales, this was the first administered Census. While the first county-level Census in New South Wales took place in 1833, adequate information on county boundaries is not available for this colony until 1834 when Surveyor General Major Thomas Mitchell was commissioned to map New South Wales into 19 formal counties. We therefore use the second New South Wales Census (which includes the Australian Capital Territory) which was held in 1836. We also use the 1842 Census in Tasmania (the first in that colony). Only the Census reports are consistently available across the relevant period, as some of the individual records were destroyed in a fire in 1882.

For all historical variables, the unit of observation is the county or police district (as applicable). Data on economic occupations comes from the Census in which it is first available (see Table A13 in the Online Appendix of Grosjean and Khattar (2018)). For a full list of maps and a description of historical data sources used in the construction of the historical variables, we refer the reader to Section 3 in that appendix.

Historical variables used in the paper

Variable	Description
Convict Sex Ratio	Number of convict men to the number of convict women
Share employed in agriculture	Proportion of population employed in agriculture
Share employed in domestic services	Proportion of population employed in domestic services
Share employed in mining and manufacturing	Proportion of population employed in mining and manufacturing

A.2 1933 Census

We take data on war service in WWI and on industrial composition from the 1933 Census of the Commonwealth of Australia, available from the Australian Bureau of Statistics.[1] The variables capturing industrial composition are built from questions on the industry of occupation of the householder at the LGA level for each state and territory (Volume I, Parts I to VI). We measure employment shares as the natural logarithm of total number of individuals employed in each industry. Variables capturing veteran status are built from the measures of the "number of males and females who served abroad with the Australian Forces in the War of 1914-191" in the LGA for each state and territory (Volume I, Parts I to VI). The proportion of male veterans is computed as the ratio of males in the LGA who served abroad with the Australian Forces in the War of 1914-1919 over the male population in the LGA. Maps of the smallest geographic unit in the 1933 Census (LGAs) are available pages 467-482 of the Statistician?s Report (Volume III). They were digitized by Grosjean and Khattar (2019). We then matched the 1933 LGAs to the historical county. Data on military records for Tasmania are taken from (Inwood et al., 2020; Cowley et al., 2021), who collected data on enlistment in the state of Tasmania. The authors collected data on 15,234 volunteers. We used the subset of 8,047 records matched to birth certificates.

A.3 Minerals, land formation, and soil quality

We take data on minerals and land formation from Geoscience Australia (https://ecat.ga.gov.au/geonetwork/srv/eng/catalog.search;jsessionid=AA779B91F9E5623DAD7B242B094803CD#/search?resultType=details&from=1&to=20&sortBy=changeDate). We downloaded topology and mineral deposits maps and aggregated this information at the post-code level.

[1]https://www.abs.gov.au/ausstats/abs@.nsf/productsbyCatalogue/9B0369AC21FF51D4CA25784C00194FA5?OpenDocument

Variable	Description
Landform	Main classification of the postcode in different categories:<br>- Plains, plateaus, sand plains<br>- Mountains
Minerals	Main classification of the postcode in different categories:<br>- Minor coal<br>- Major gold<br>- No minerals or traces

Data on soil quality comes from the Global Agro-ecological zones assessment. We include the variables:

- Soil: Toxicity Soil: Excess salts
- Soil: Oxygen availability to roots
- Soil: Nutrient retention capacity
- Soil: Nutrient availability
- Mapped water bodies (% postcode)

which represent the average of the class over postcode area for a 5′ latitude by 5′ longitude grid-cell. Classes are the following: 1: No or slight limitations 2: Moderate limitations 3: Sever limitations 4: Very severe limitations 5: Mainly non-soil 6: Permafrost area 7: Water bodies

Source: Fischer, G., F. Nachtergaele, S. Prieler, H.T. van Velthuizen, L. Verelst, D. Wiberg, 2008. Global Agro-ecological Zones Assessment for Agriculture (GAEZ 2008). IIASA, Laxenburg, Austria and FAO, Rome, Italy. http://www.fao.org/soils-portal/data-hub/soil-maps-and-databases/harmonized-world-soil-database-v12/en

For mean annual temperature in 1902, data are from University of East Anglia Climatic Research Unit; Jones, P.D.; Harris, I.C. (2008): Climatic Research Unit (CRU): Time-series (TS) datasets of variations in climate with variations in other phenomena v3. NCAS British Atmospheric Data Centre, 27/07/2021. https://www.globalclimatemonitor.org/# Mean temperature over the postcode on the 0.5x0.5 degree grid. TIFF band values are classified on 1-255 scale where the values have been recoded such that higher values correspond to higher temperature.

A.4 Census

We use the following SA1-level controls from the 2011 and 2016 Australian Census. The variables are constructed by averaging the values across both census waves. We also use the 2011 and 2016 Australian Census to construct employment shares by gender and occupation type (again, these are averages across both waves of the census). Employment by occupation (at the 4-digit level) is at the postcode level instead of SA1-level, due to small cell sizes and censoring at the SA1-level.

Census variables from 2011 and 2016 (SA1 level)

Variable	Description
	Main controls
Contemporary sex ratio	Number of men to the number of women
Contemporary population	Total population
Population density	Total population in SA1 divided by total land area of SA1
Urban	Dummy variable equal to one if SA1 is classified as urban by the Australian Bureau of Statistics
	Extended controls
Unemployment rate (by gender)	Percentage of people not working more than one hour in the reference week; actively looking for work in previous four weeks; and being available to start work in the reference week.
Religious shares	% of the population self-declaring as:<br>- Buddhist<br>- Anglican<br>- Catholic<br>- Other Christian<br>- Islam<br>- No religion
Median age	Median age of persons in SA1
Percent completed high school	Percentage of people who completed year 12 education (graduated from high school)
Percent foreign born	Percentage of the population born outside of Australia
Median household weekly income	Median total household weekly income (calculated by the ABS)

Occupational gender segregation: 2011 and 2016 Census (postcode level)

Variable	Description
Share of men/women in feminine/masculine/neutral occupations	We first classify occupations into three groups (feminine/masculine/neutral). To ensure that we pick up occupations that are known to be "stereotypically male/female", we classify the most common occupations at the 4-digit level (occupations with total employment shares greater than 0.5%, approximately 55 of a total of 469 occupations, with 55% of the workforce represented in these occupations). Of the common occupations, they are then considered feminine/neutral/masculine if their national male share in the occupation is less than 33% (feminine), between 33-66% (neutral), or more than 66% (masculine). To compute the share of men in feminine/masculine/neutral occupations employed in a given postcode, we calculate the percent of men (of total men employed in a given postcode) that are employed in each of the three categories of occupations. This is done analogously for women.
Total masculine or feminine occupations	Total employed in most extreme male/female common occupations (defined as having 85% or more of one gender, employed nationally) in the postcode. Included as a control, log-transformed.

A.5 Violence and crime data

We obtain crime data by postcode for each state. Australian states are separate criminal jurisdictions and crime classification and reporting therefore varies. For New South Wales crime data is publicly available from dedicated statistical agencies (the NSW Bureau of Crime Statistics and Research). Data was obtained from the Tasmanian Department of Police after filing a special request. In the Australian Capital Territory additional procedures and filing of a Freedom of Information act are necessary.

Violence and crime data available in Australia

State	Type of crime reported	Reporting years
NSW	- Homicide<br>- Assaults (broken down by assault against police, domestic violence, non-domestic violence)<br>- Sexual offenses<br>- Robbery<br>- Theft<br>- Drug offenses<br>- Disorderly conduct (with several subcategories)<br>- Other offences	1995–2016
TAS	- Homicide<br>- Assaults<br>- Sexual assault<br>- Offences against property	1999–2016

We only retain data between 2006 and 2016. We merge these crime data with early counts of the population from the 2006, 2011, and 2016 Censuses. We interpolate in between Census years to compute rates of assaults per 100,000 people. Below is a description of the variables used in the paper and information on the available data:

Violence and crime variables used in the paper

Variable	Description
Assault	Natural logarithm of the mean of the number of all assaults per 100,000 people between 2006 and 2016 (+1)
Sexual offenses	Natural logarithm of the mean of the number of all domestic assaults per 100,000 people between 2006 and 2016 (+1)
Property crime	Natural logarithm of the mean of the number of all robbery and theft/offences against property per 100,000 people between 2006 and 2016 (+1)

A.6 Mortality

We use the data set Mortality over Regions and Time 2011-2015 as published by Australian Government's Australian Institute of Health and Welfare. These data are available to download here. The data set lists the top 20 causes of death by gender and Local Government Area (LGA) over this time period, as well as the total number of deaths in each year. We generated the following variables by LGA and gender, and then merged to the historical counties by matching LGAs to 2011 postcodes using ABS correspondence tables.

All death variables used as outcomes are transformed such that we use log of male (or female) deaths per 100,000 males (or females) in the LGA. Below is a description of the variables used in the paper and details on how the variables were constructed:

Mortality variables used in the paper

Variable	Description
Total deaths	Average number of total deaths due to all causes between 2011-2015. The total number of deaths is reported for each year between 2011 and 2015, and we take the average over this period. Log-total deaths is used as a control to adjust for the age distribution over this particular period in a particular locality.
Suicide (male only)	Number of deaths due to suicide. We report results for males only because suicide appears in the top 20 causes of death approximately 20 percent of the time for females. For females, we only report a binary variable indicating that the LGA reports suicide as a top-20 cause of death for females.
Prostate cancer (male only)	Number of deaths due to prostate cancer and other conditions related to male genital organs. Causes of death attributed to prostate cancer and other conditions related to male genital organs includes diseases of male genital organs; malignant neoplasms of penis, testis, other male genital organs; prostate cancer.

A.7 Referendum on same-sex marriage

The Australian Marriage Law Postal Survey was conducted by the Australian Bureau of Statistics (ABS) as a postal vote between 12 September and 7 November 2017. Turnout was 79.5 percent. The results of the referendum were released at the Federal Electoral Division level (150 Federal Electoral Divisions) by the ABS on 15 November 2017 (abs.gov.au/ausstats/abs@.nsf/mf/1800.0) and accessed by the researchers on 15 November 2017 at 7PM.

A.8 HILDA

HILDA is an Australian nationally representative survey available since 2001 on an annual basis (with the set of variables changing across years). We use data from the waves 2011 and 2015. HILDA provides a vast array of information on households and individuals across Australia.

Same-sex marriage referendum vote

Variable	Description
% voted 'Yes'	Percentage of total eligible registered voters who voted 'Yes' to the question posed in the Marriage Law Postal Survey: "*Should the law be changed to allow same-sex couples to marry?*"
% abstention	Percentage of total eligible registered voters who did not send back their reply in the Marriage Law Postal Survey

For all HILDA variables, the unit of observation is an individual living in an SA1.

HILDA survey variables

Variable	Description
Supports same-sex marriage	A dummy variable taking value 1 if the respondents' answer to the following question: *"How much do you agree with the statement: 'Homosexual couples should have the same rights as heterosexual couples do'"* is strictly above 3. Response categories range from 1 (strongly disagree) to 7 (strongly agree).

A.9 LSAC

The Longitudinal Study of Australian Children (LSAC) is a major study following the development of 10,000 young people and their families from all parts of Australia. The study began in 2003 with a representative sample of children (who are now teens and young adults) from urban and rural areas of all states and territories in Australia. Data are collected from two cohorts every two years. The first cohort of 5,000 children was aged 0-1 years in 2003-04, and the second cohort of 5,000 children was aged 4-5 years in 2003-04. Study informants include the young person, their parents (both resident and non-resident), carers and teachers. We use both cohorts of data over seven waves between 2004 and 2016 (with ages between 4 and 15). The unit of observation is a child living in a postcode during the wave/year of data collection.

LSAC survey variables

Variable	Description
Child experienced bullying, reported by parents	A dummy variable taking value 1 if either parent reported that their child experienced bullying
Child experienced bullying, reported by teacher	A dummy variable taking value 1 if the teacher reported that the child experienced bullying
Child experienced bullying, reported by child	A dummy variable taking value 1 if the child self-reported to have experienced bullying. This variable was not used because the sample is much smaller as it was only asked of children aged 10 or above.

A.10 Election Voting

Electoral voting data is based on the first-preferences of the Senate votes by polling place in the 2016 election. We thank Haishan Yuan for sharing this data, matched to more than 8300 polling places in Australia, based on the raw data from the Australian Electoral Commission.

http://results.aec.gov.au/20499/Website/HouseDownloadsMenu-20499-Csv.htm

A.11 Vaccine Hesitancy

Vaccine hesitancy comes from the Taking the Pulse of the Nation survey. (Taking the Pulse of the Nation survey (2021), Melbourne Institute: Applied Economic & Social Research). The question on vaccine hesitancy was asked in three waves: (05oct2020 - 10oct2020) "If a vaccine for COVID-19 is developed and approved for use by the Australian Government, would you be willing to be vaccinated?"; (01feb2021 - 05jun2021) "Are you willing to have the covid - 19 vaccine?"; (14jun2021 - 23sep2021) This wave asked the previous question with option to answer "I have had the FIRST dose of the vaccine ONLY" or "I have had the first AND second dose of the vaccine". A total of 2595 male and 2571 female respondents were surveyed from the historical counties analyzed in this paper. Data were provided as counts (e.g. the total number of respondents endorsing the statement) at the postcode level for the age group 18-74, dis-aggregated by gender. These were merged to the historical data at the SA1 level (a finer mesh).

References

Cowley, Trudy, Lucy Frost, Kris Inwood, Rebecca Kippen, Hamish Maxwell-Stewart, Monika Schwarz, John Shepherd, Richard Tuffin, Mark Williams, John Wilson, et al. 2021. "Reconstructing a longitudinal dataset for Tasmania." *Historical Life Course Studies*, 11(2): 1–30.

Inwood, Kris, Rebecca Kippen, Hamish Maxwell-Stewart, and Richard Steckel. 2020. "The short and the tall: comparing stature and socioeconomic status for male prison and military populations." *Social Science History*, 44(3): 463–483.
